# Perturbational Analysis of Magnetic Force Theorem for Magnetic Exchange Interactions in Molecules and Solids

**DOI:** 10.3390/molecules29215190

**Published:** 2024-11-02

**Authors:** Dong-Kyun Seo

**Affiliations:** School of Molecular Sciences, Arizona State University, Tempe, AZ 85287-1604, USA; dseo@asu.edu

**Keywords:** spinors, noncollinear magnetism, RKKY interactions, magnetic force theorem, Lichtenstein formula, Brillouin–Wigner perturbation method, Green’s function, magnetic exchange interactions, linear response theory, density functional perturbation theory

## Abstract

There have been increasing efforts to compute magnetic exchange coupling constants for transition metal complexes and magnetic insulators using the magnetic force theorem and Green’s function-based linear response methods. These were originally conceived for magnetic metals, yet it has not been clear how these methods fare conceptually with the conventional models based on electron-correlation interactions among so-called magnetic orbitals. We present a spinor-based theoretical analysis pertinent to the magnetic force theorem and linear response theory using Brillouin–Wigner perturbation method and Green’s function perturbation method, and we shed light on the conceptual nature of the Lichtenstein formula in its applications for calculations of the total energy and magnetic exchange coupling constants for both molecules and solids. Derivation of the magnetic force theorem in this perturbational analysis identifies the first-order energy correction terms, which are considered as the ferromagnetic component for the magnetic exchange interactions of transition metal compounds but are not included in the Lichtenstein formula. Detailed perturbational analysis of the energy components involved in the magnetic force theorem identifies the energy components that are missing in the Lichtenstein formula but are critical in the Anderson’s model for transition metal complexes and magnetic insulators where magnetic orbitals can overlap.

## 1. Introduction

The magnetic exchange coupling constant $J_{\mathrm{ab}}$ is the coefficient of the scalar product between the spin vectors of two magnetic sites, say ***a*** and ***b***, in the Heisenberg–Dirac–Van Vleck (HDVV) Hamiltonian, which is modeled to describe the magnetic exchange interactions between the following two sites:

(1) $$\begin{array}{c} H^{HDVV}=-J_{\mathrm{ab}}{\vec{S}}_{a}\cdot {\vec{S}}_{b}=-J_{\mathrm{ab}}S_{a}S_{b}\cos\theta \;. \end{array}$$

The sinusoidal form of the HDVV Hamiltonian indicates that the $J_{\mathrm{ab}}$ value can be obtained as the second derivative of the change in the total energy ($\Delta E_{\mathrm{tot}}$) from a reference state or the total energy $E_{\mathrm{tot}}$ itself with respect to the angle θ between the spin directions of the two sites:

(2) $$\begin{array}{ccc} J_{\mathrm{ab}} & = & -\frac{1}{S_{a}S_{b}}{\left[\frac{\partial^{2}\Delta E_{\mathrm{tot}}}{\partial \theta^{2}}\right]}_{0}=-\frac{1}{S_{a}S_{b}}{\left[\frac{\partial^{2}E_{\mathrm{tot}}}{\partial \theta^{2}}\right]}_{0}\;. \end{array}$$

The second identity in the expression is obtained by dropping the fixed total energy term of the reference state. The HDVV model is the simplest model that works for various magnetic systems, regardless of the nature of the magnetic exchange interactions.

In his seminal monograph ”Magnetism and the Chemical Bond”, published in 1963, Goodenough provided an extensive review of the then state-of-the-art knowledge concerning the origins of atomic moments and of magnetic exchange interactions in solids [1]. While most of the book was on ionic compounds of the transition metal elements, the book also provided qualitative ideas on the magnetism of metals and alloys, where collective *d* electrons coexist with conducting *s* and *p* electrons, with a postulation that the configuration interaction is appropriate for localized *d* electrons. This gives the superexchange rules and the Anderson’s magnetic orbital model, which remains the principal configuration interaction responsible for electron correlations among collective *d* electrons. What was notably missing in the book was remarks on another type of magnetism in magnetic metals; that is, the indirect magnetic exchange interactions between localized magnetic moments through itinerant electrons (“Ruderman–Kittel–Kasuya–Yosida (RKKY) interactions”) [2,3].

In RKKY theory, when a localized moment is introduced into a metal, the conduction spins develop an oscillating polarization (called “Friedel oscillations” in the case of free electrons) in the vicinity of this moment. Quantum mechanical estimation of the magnetic exchange coupling constants for the RKKY interactions has been possible for real materials through combined application of magnetic force theorem and linear response theory within the framework of Korringa—Kohn—Rostoker (KKR) multiple-scattering theory, which culminates in the Lichtenstein formula [4]. While the original derivation of the formula relied on the use of the Lloyd formula in multiple scattering theory, recent studies have provided different expressions that employ atomic orbitals as the fundamental entities in magnetic exchange interactions [5]. Such developments have motivated quantitative applications of the Lichtenstein formula for multinuclear transition metal complexes and Mott–Hubbard magnetic insulators with various computational methods as an alternative way of calculating the magnetic exchange coupling constants for those compounds [6,7,8,9,10,11,12,13].

However, the notion that the RKKY interaction is mediated by the electrons that can be described by single-particle electronic band structures is quite in contrast to the nature of the magnetic exchange interactions in Anderson’s model in which electron correlations among neighboring localized magnetic centers play a dominant role [14,15,16]. Therefore, the validity of the application of the Lichtenstein formula to Mott–Hubbard magnetic insulators has not been established from a theoretical point of view, especially regarding how it is compared to Anderson’s model in terms of the fundamental concepts and formalisms. In contrast to the latter, which takes advantage of the concept of magnetic orbitals, it is quite challenging to visualize orbital interactions for delocalized electrons to the extreme of the oscillatory spin polarization predicted from free electron theory.

Herein, we present a detailed analysis of the density functional energy components that occur in the magnetic force theorem and the Lichtenstein formula by deriving the theorem and formula using a perturbational approach that is more conventional in the studies of magnetic insulators. The analysis is based on our previous work that has been successful in understanding the density functional results using magnetic orbitals within the broken-symmetry approach [16]. The method has also allowed for a unified view on different concepts such as Stoner condition and RKKY-type induced spin polarization, together with Anderson’s model, at least within the limits of the collinear magnetism [17].

This work is limited to a theoretical evaluation and rationalization of the existing methods rather than case studies with computations of the magnetic exchange coupling constants for real examples. The derivations of the mathematical expressions are shown in great detail. Although they might be exhaustive, the intention is to avoid a pitfall of the previous works, where a large part of the mathematical derivations is missing, leading to incomprehension or misinterpretation. In particular, the assumptions and approximations are stated clearly wherever they are used, with justifications. Some of the perturbational expressions given in this work are for the qualitative analysis of the computational results, which can provide intuitive guidelines in designing new magnetic materials. The novel spinor-based approach can be used to provide a new semi-quantitative way of understanding the effects of spin-orbit coupling on noncolinear magnetism, which will be published separately in the near future.

This work is organized as follows: First, in Section 2.1, we expand our original perturbational method to noncollinear magnetism by finding approximate expressions for the exchange-correlation (xc) functional and consequently by linearizing the xc potential using a vector representation of the spin polarization. Second, in Section 2.2, we examine the spinor-based Kohn–Sham equation and identify the expressions and relationships of the essential components in our perturbational method, including spinors, spinor energies, electronic structure energy, and double-count energy for both unperturbed and perturbed magnetic states and thus their total energy difference. Furthermore, in Section 2.2, we utilize the approximate expressions of the energy terms and examine the nature of magnetic force theorem within the framework of density-functional perturbation theory. This allows us to find a general expression for the total energy change upon a small change in spin density as a perturbation in a magnetic state. In particular, it identifies what the reference state should be like in applying the magnetic force theorem to obtain the total energy change by a perturbation and eventually the magnetic exchange coupling constant.

Third, in Section 2.2, we first describe the proper selection of the zeroth-order spinors and introduce an intermediate normalization condition that is advantageous in simplifying the expressions for spinors and spinor energies of the perturbed state. Then, explicit expressions for the perturbed spinors and spinor energies are obtained in Section 2.2.3 by employing the Brillouin–Wigner perturbation method. The summation of the perturbed spinor energies over the occupied spinors provide the expressions for the electronic structure energy change and ultimately for the total energy change after applying the magnetic force theorem. In Section 2.2.4, we use the magnetic force theorem and Green’s function perturbation theory to derive the expression for the total energy change for continuous eigenvalue systems and show that the expressions from the Brillouin–Wigner and Green’s function perturbation methods are equivalent.

Lastly, in Section 2.3, we use two simple examples to illustrate the meaning of various energy terms in $\Delta E_{\mathrm{tot}}$. With a simple magnetic dimer having one magnetic orbital on each site, we derive the ferromagnetic and antiferromagnetic terms in the magnetic exchange coupling constant in Anderson’s model. The same expressions have been reported previously within the density functional theory, but the method was limited to the case of collinear magnetism, where the the magnetic exchange coupling constants are obtained from the total energy differences between ferromagnetic and antiferromagentic states [16]. In contrast, the spinor approach employed in this work is consistent with the spin vector interactions in the HDVV Hamiltonian and thus provides direct insight into how the HDVV-type interaction term can manifest even in the single-electron theory. For RKKY interactions, a simple free electron model is employed to show how Lichtenstein’s recipe works for the systems where localized magnetic moments interact solely via conducting electrons. It is further indicated what energy components are missing in the Lichtenstein formula to correctly describe the magnetic interactions in the magnetic insulators.

## 2. Results

### 2.1. Presets for Perturbational Analysis

#### 2.1.1. Kohn–Sham Hamiltonian and Total Energy Components

A general description of magnetic exchange interactions should be able to treat the situations where the spin quantization axes are not linear among the interacting magnetic sites (*noncollinear magnetism*) [18]. The noncollinear magnetic system is described by 2×2 Kohn–Sham matrix equations for spinor wavefunctions, $\psi_{i}$s, and their eigenvalues, $\epsilon_{i}$’s [18]:

(3) $$\begin{array}{c} H\psi_{i}=\epsilon_{i}\psi_{i}\;, \end{array}$$

where the eigenspinor $\psi_{i}$ has a spatial component $\psi_{i}$ and a unit spinor $\omega_{i}$ as a spin component with its spin orientation along a unit vector $u_{i}$ (i.e., $\omega_{i}^{†}\sigma \omega_{i}=u_{i}$):

(4) $$\begin{array}{ccc} \psi_{i} & = & \psi_{i}\omega_{i}\;. \end{array}$$

Each magnetic state is described uniquely by the total electron density, ρ(r), as the sum of all the electron densities among the occupied spinors, $\psi_{i}$’s ($\rho_{i}$):

(5) $$\begin{array}{c} \rho \left(r\right)=\sum_{i}\;n_{i}\rho_{i}\left(r\right)\;, \end{array}$$

where the spinor electron density of $\psi_{i}$ is given with the help of $\omega_{i}^{†}\omega_{i}=1$ as follows:

(6) $$\begin{array}{c} \rho_{i}\left(r\right)=\psi_{i}^{†}\left(r\right)\psi_{i}\left(r\right)=\psi_{i}^{*}\left(r\right)\psi_{i}\left(r\right)\left(\omega_{i}^{†}\omega_{i}\right)={\left|\psi_{i}\left(r\right)\right|}^{2}\;, \end{array}$$

and by the total spin density vector, ${\vec{\rho }}_{S}\left(r\right)$, as the sum of all the spinor spin densities (${\vec{\rho }}_{Si}$) over all the occupied spinors:

(7) $$\begin{array}{c} {\vec{\rho }}_{S}\left(r\right)=\sum_{i}\;n_{i}{\vec{\rho }}_{Si}\left(r\right)\;, \end{array}$$

where the spinor spin density of $\psi_{i}$ is the multiplication product of the spinor electron density and the spin unit vector:

(8) $$\begin{array}{c} {\vec{\rho }}_{Si}\left(r\right)=\psi_{i}^{†}\left(r\right)\vec{\sigma }\psi_{i}\left(r\right)=\psi_{i}^{*}\left(r\right)\psi_{i}\left(r\right)\left(\omega_{i}^{†}\sigma \omega_{i}\right)=\rho_{i}\left(r\right)u_{i}\;. \end{array}$$

The symbol $n_{i}$ is the occupancy number, 0 or 1, for $\psi_{i}\left(r\right)$. The mathematical symbol (r) will often be dropped in the expressions of r-dependent functions for the sake of simplicity throughout this work. The symbol $n_{i}$ is the occupancy number, 0 or 1, for $\psi_{i}\left(r\right)$. The mathematical symbol (r) will often be dropped in the expressions of r-dependent functions for the sake of simplicity throughout this work. The total density ρ and the total spin density ${\vec{\rho }}_{S}$ are the two components of the one-electron density matrix ρ:

(9) $$\begin{array}{ccc} \rho & = & \sum_{i}\;n_{i}\psi_{i}\psi_{i}^{†}=\frac{1}{2}\left(\rho \sigma_{0}+{\vec{\rho }}_{S}\cdot \vec{\sigma }\right)\;, \end{array}$$

where $\sigma_{0}$ and $\vec{\sigma }$ are the 2×2 identity matrix and the Pauli vector ($\sigma_{x},\sigma_{y},\sigma_{z}$), respectively.

The total electronic energy is given as the electronic structure (“e.s.”) energy ($E_{e.s.}$) corrected by the double-count (“d.c.”) energy ($E_{d.c.}$):

(10) $$\begin{array}{c} E_{\mathrm{tot}}=E_{e.s.}+E_{d.c.}\left[\rho ,{\vec{\rho }}_{S}\right]\;, \end{array}$$

with the electronic structure energy defined as the sum of the spinor energies over all the occupied spinors:

(11) $$\begin{array}{c} E_{e.s.}=\sum_{i}\;n_{i}\epsilon_{i}\;. \end{array}$$

The Kohn–Sham Hamiltonian is given as follows:

(12) $$\begin{array}{c} H=\left(-\frac{1}{2}\nabla^{2}+V_{ext}\left(r\right)+V_{H}\left(r\right)\right)\sigma_{0}+V_{\mathrm{xc}}\left(r\right)\;, \end{array}$$

in atomic units ($\hbar =m_{e}=\mu_{B}=-e=1$), where $V_{ext}$ and $V_{H}$ are the nuclear–electron attraction potential and the Hartree potential, respectively [18]. The xc potential matrix $V_{\mathrm{xc}}$ is obtained from the xc energy $E_{\mathrm{xc}}\left[\rho ,{\vec{\rho }}_{S}\right]$ as follows:

(13) $$\begin{array}{c} V_{\mathrm{xc}}\left(r\right)=V_{\mathrm{xc}}^{\mathrm{nsp}}\left(r\right)\sigma_{0}+v^{S}\left(r\right)\cdot \vec{\sigma }\;, \end{array}$$

with the definitions of

(14) $$\begin{array}{c} V_{\mathrm{xc}}^{\mathrm{nsp}}\left(r\right):=\frac{\partial E_{\mathrm{xc}}\left[\rho ,{\vec{\rho }}_{S}\right]}{\partial \rho }\;,\;v^{S}\left(r\right):=\frac{\partial E_{\mathrm{xc}}\left[\rho ,{\vec{\rho }}_{S}\right]}{\partial {\vec{\rho }}_{S}}\;. \end{array}$$

Here, $V_{\mathrm{xc}}^{\mathrm{nsp}}$ and $v^{S}\left(r\right)\cdot \vec{\sigma }$ are spin-independent and spin-dependent components of the xc potential, respectively. With Equation (13), the Hamiltonian in Equation (12) can be re-expressed as follows:

(15) $$\begin{array}{c} H=H^{\mathrm{nsp}}+v^{S}\left(r\right)\cdot \vec{\sigma }\;, \end{array}$$

where $H^{\mathrm{nsp}}$ is the spin-independent part containing the kinetic energy operator and $V_{ext}$, $V_{H}$, and $V_{\mathrm{xc}}^{\mathrm{nsp}}$:

(16) $$\begin{array}{c} H^{\mathrm{nsp}}=\left(-\frac{1}{2}\nabla^{2}+V_{ext}\left(r\right)+V_{H}\left(r\right)+V_{\mathrm{xc}}^{\mathrm{nsp}}\left(r\right)\right)\sigma_{0}\;, \end{array}$$

while $v^{S}\cdot \vec{\sigma }$ is the spin-dependent potential matrix. The vector $v^{S}$ would interact with the spinors vectorially in the form of $v^{S}\cdot \vec{\sigma }$. The meaning of this mathematical form will become clear in later sections. Using Equations (8) and (15), the eigenvalue $\epsilon_{i}$ of the spinor $\psi_{i}$ is also divided into the two parts, one with spin-independent energy terms of the Hamiltonian and the other with the spin-dependent term:

(17) $$\begin{array}{ccc} \epsilon_{i} & = & \langle \psi_{i}|H|\psi_{i}\rangle =\langle \psi_{i}|H^{\mathrm{nsp}}|\psi_{i}\rangle +\int v^{S}\cdot {\vec{\rho }}_{Si}\;dr\;. \end{array}$$

From Equations (7), (11) and (17), the electronic structure energy is decomposed in the same way:

(18) $$\begin{array}{c} E_{e.s.}=\sum_{i}n_{i}\langle \psi_{i}|H^{\mathrm{nsp}}\psi_{i}\rangle +\int v^{S}\cdot {\vec{\rho }}_{S}\;dr\;. \end{array}$$

Since the spinor eigenfunctions ($\psi_{i}$’s) are spin-dependent, so are the first energy terms in Equations (17) and (18), although they contain only the spin-independent part of the Hamiltonian. The later work in Section 2.2 will provide the explicit expressions for the spin-dependent part of $\psi_{i}$s.

The second term in Equation (17) describes the interaction of the spinor with the total spin density as their scalar product. Summation of this over all the occupied spinors leads to the second term in Equation (18). This term is also present in the double-count energy $E_{d.c.}$ (Equation (10)), which is composed of the potential energy terms only:

(19) $$\begin{array}{c} E_{d.c.}\left[\rho ,{\vec{\rho }}_{S}\right]=-E_{H}\left[\rho \right]+E_{\mathrm{xc}}\left[\rho ,{\vec{\rho }}_{S}\right]-\int V_{\mathrm{xc}}^{\mathrm{nsp}}\rho \;dr-\int v^{S}\cdot {\vec{\rho }}_{S}\;dr\;, \end{array}$$

where $E_{H}\left[\rho \right]$ is the Hartree energy, which is spin-independent. The spin-dependent component of the double-count energy is given as follows:

(20) $$\begin{array}{ccc} E_{d.c.}^{S}\left[\rho ,{\vec{\rho }}_{S}\right] & = & E_{\mathrm{xc}}\left[\rho ,\rho_{S}\right]-E_{\mathrm{xc}}\left[\rho ,0\right]-\int v^{S}\cdot {\vec{\rho }}_{S}\;dr\;. \end{array}$$

Since we assume negligible differences in the electron densities among different magnetic states, the change in the total electronic energies (Equation (10)) contains only the spin-dependent energy components:

(21) $$\begin{array}{ccc} \Delta E_{\mathrm{tot}} & \approx & \Delta E_{e.s.}+\Delta E_{d.c.}^{S} \end{array}$$

#### 2.1.2. Linearization of xc Potential in Noncollinear Magnetism

In all our discussions, the xc energy $E_{\mathrm{xc}}\left[\rho ,{\vec{\rho }}_{S}\right]$, a functional of both ρ and ${\vec{\rho }}_{S}$, is a key feature. One important assumption to make is that the electron density does not change significantly among different magnetic states [16,19]. Therefore, different magnetic states are defined by their corresponding different spin densities upon the assumption of negligible changes in the electron density. *The perturbation of our interest is represented solely by a small change in spin density, since the effects of the total electron density change on the total electronic energy are assumed to be negligible*.

In order to examine the role of the spin density in $E_{\mathrm{xc}}\left[\rho ,{\vec{\rho }}_{S}\right]$, we separate the spin-dependent component using the Taylor expansion of the the xc energy in ${\vec{\rho }}_{S}$ [16,19]. By taking into account the time-reversal symmetry of the xc energy (i.e., $E_{\mathrm{xc}}\left[\rho ,-{\vec{\rho }}_{S}\right]=E_{\mathrm{xc}}\left[\rho ,{\vec{\rho }}_{S}\right]$), the xc energy is approximated as follows:

(22) $$\begin{array}{ccc} E_{\mathrm{xc}}\left[\rho ,{\vec{\rho }}_{S}\right] & \approx & E_{\mathrm{xc}}\left[\rho ,0\right]-\frac{1}{4}\;\int \int \;{\vec{\rho }}_{S}\left(r^{\prime }\right)\cdot K_{\mathrm{xc}}\left(r,r^{\prime }\right)\cdot {\vec{\rho }}_{S}\left(r\right)dr^{\prime }dr\;, \end{array}$$

where $K_{\mathrm{xc}}\left(r,r^{\prime }\right)$, a 3×3 tensor, is spin-independent and called the “spin hardness function” [18,19]:

(23) $$K_{\mathrm{xc}}\left(r,r^{\prime }\right):=-2{\left[\frac{\partial^{2}E_{\mathrm{xc}}\left[\rho ,{\vec{\rho }}_{S}\right]}{\partial {\vec{\rho }}_{S}\left(r^{\prime }\right)\partial {\vec{\rho }}_{S}\left(r\right)}\right]}_{0}\;.$$

The approximation in Equation (22) is accurate up to the third order in ${\vec{\rho }}_{S}$, because all the odd-order terms are zero due to the time-reversal symmetry. Within the local spin density (LSD) approximation, the exchange correlation energy expression in Equation (22) becomes as follows:

(24) $$\begin{array}{c} E_{\mathrm{xc}}^{\mathrm{LSD}}\left[\rho ,\rho_{S}\right]\approx E_{\mathrm{xc}}^{\mathrm{LSD}}\left[\rho ,0\right]-\frac{1}{4}\int \kappa_{\mathrm{xc}}\left(r\right){\vec{\rho }}_{S}\left(r\right)\cdot {\vec{\rho }}_{S}\left(r\right)\;dr=E_{\mathrm{xc}}^{\mathrm{LSD}}\left[\rho ,0\right]-\frac{1}{4}\int \kappa_{\mathrm{xc}}\left(r\right)\rho_{S}{\left(r\right)}^{2}dr\;, \end{array}$$

where $\kappa_{\mathrm{xc}}\left(r\right)$ is called the “local spin hardness function” and is always *positive*:

(25) $$\kappa_{\mathrm{xc}}\left(r\right):=-2{\left[\frac{\partial^{2}E_{\mathrm{xc}}^{\mathrm{LSD}}\left[\rho ,\rho_{S}\right]}{\partial \rho_{S}{\left(r\right)}^{2}}\right]}_{0}\;.$$

Meanwhile, the approximate xc potential is obtained from Equation (22) using Equation (14):

(26) $$\begin{array}{cc} V_{\mathrm{xc}}\left(r\right) & \approx \frac{\partial E_{\mathrm{xc}}\left[\rho \left(r\right),0\right]}{\partial \rho (r)}\sigma_{0}-\left(\frac{1}{2}\int {\vec{\rho }}_{S}\left(r^{\prime }\right)\cdot K_{\mathrm{xc}}\left(r,r^{\prime }\right)\;dr^{\prime }\right)\cdot \vec{\sigma }\\ V_{\mathrm{xc}}^{\;\mathrm{LSD}}\left(r\right) & \approx \frac{\partial E_{\mathrm{xc}}^{\mathrm{LSD}}\left[\rho \left(r\right),0\right]}{\partial \rho (r)}\sigma_{0}-\frac{1}{2}\kappa_{\mathrm{xc}}\left(r\right){\vec{\rho }}_{S}\left(r\right)\cdot \vec{\sigma }\;. \end{array}$$

The scalar quantity in the first terms in Equation (26) is $V_{\mathrm{xc}}^{\mathrm{nsp}}$, while the spin-dependent vector in the second terms corresponds to $v^{S}$, a spin polarization vector:

(27) $$\begin{array}{ccc} v^{S}\left(r\right) & \approx & -\frac{1}{2}\int {\vec{\rho }}_{S}\left(r^{\prime }\right)\cdot K_{\mathrm{xc}}\left(r,r^{\prime }\right)\;dr^{\prime }\overset{\mathrm{LSD}}{\approx }-\frac{1}{2}\kappa_{\mathrm{xc}}\left(r\right){\vec{\rho }}_{S}\left(r\right)\;. \end{array}$$

It is important to note that the spin-dependent part $v^{S}$ in the xc potential is *negatively* proportional to the spin vector ${\vec{\rho }}_{S}$ after the linearization.

With the approximations above, the spin-dependent part of the double-count energy in Equation (20) is simplified as follows:

(28a)Ed.c.S[ρ,ρ→S]≈−12∫vS(r)·ρ→S(r)dr(28b)≈14∫∫ρ→S(r′)·Kxc(r,r′)·ρ→S(r)dr′dr(28c)≈LSD14∫κxc(r)ρS2(r)dr.

Using Equations (18), (19), and (28), we find that the total electronic energy in Equation (10) can be re-expressed to identify two spin-dependent terms, $\sum_{i}n_{i}\langle \psi_{i}|H^{\mathrm{nsp}}|\psi_{i}\rangle$ and $-E_{d.c.}^{S}\left[\rho ,{\vec{\rho }}_{S}\right]$, and two spin-independent terms, $-E_{H}\left[\rho \right]$ and $E_{\mathrm{xc}}\left[\rho ,0\right]$:

(29) $$\begin{array}{ccc} E_{\mathrm{tot}} & \approx & \sum_{i}n_{i}\langle \psi_{i}|H^{\mathrm{nsp}}|\psi_{i}\rangle -E_{H}\left[\rho \right]+E_{\mathrm{xc}}\left[\rho ,0\right]-E_{d.c.}^{S}\left[\rho ,{\vec{\rho }}_{S}\right]\;. \end{array}$$

In Equation (29), $-E_{d.c.}^{S}$ is often called the “spin polarization energy” ($E_{\mathrm{sp}}$):

(30) $$\begin{array}{ccc} E_{\mathrm{sp}}\left[\rho ,{\vec{\rho }}_{S}\right] & = & -E_{d.c.}^{S}\approx \frac{1}{2}\int v^{S}\cdot {\vec{\rho }}_{S}\;dr\overset{\mathrm{LSD}}{\approx }-\frac{1}{4}\int \kappa_{\mathrm{xc}}\;\rho_{S}^{2}\;dr\;, \end{array}$$

since it is the the component of the total electronic energy that is solely dependent of the total spin (polarization) density. Thus, when we denote the sum of all the spin-dependent components in Equation (29) as $E_{\mathrm{tot}}^{S}$, we find the following:

(31) $$\begin{array}{ccc} E_{\mathrm{tot}}^{S} & \approx & \sum_{i}n_{i}\langle \psi_{i}|H^{\mathrm{nsp}}|\psi_{i}\rangle +E_{\mathrm{sp}}\;, \end{array}$$

and obtain an expression of $J_{\mathrm{ab}}$ alternative to Equation (2):

(32) $$\begin{array}{ccc} J_{\mathrm{ab}} & \approx & -\frac{1}{S_{a}S_{b}}{\left[\frac{\partial^{2}\Delta E_{\mathrm{tot}}^{S}}{\partial \theta^{2}}\right]}_{0}=-\frac{1}{S_{a}S_{b}}{\left[\frac{\partial^{2}E_{\mathrm{tot}}^{S}}{\partial \theta^{2}}\right]}_{0}. \end{array}$$

In other words, when the electron densities are similar among different magnetic states, what we would need for the calculation of $J_{\mathrm{ab}}$ is the mathematical expression that relates $E_{\mathrm{tot}}^{S}$ to the angle θ between the spin vectors of the interacting magnetic sites. However, the expression of $E_{\mathrm{tot}}^{S}$ in Equation (31) is not practical in general for the calculation of $J_{\mathrm{ab}}$, and the applicable expressions will be derived at the endings of Section 2.2.1 and Section 2.2.2 based on the magnetic force theorem and the perturbational analysis of the energies.

#### 2.1.3. Magnetic Force Theorem

Before we proceed to perturbational treatment of the spinors, we rederive the *magnetic force theorem*, as this theorem helps us find proper expressions for the total electronic energy change when a magnetic system transitions to a new magnetic state with a different sin density. The Andersen’s local force theorem states that within local density functional theory, the total energy change in an electronic system due to a small perturbation is to the first order the change in the electronic structure energy that is calculated by fixing the electron density [20]. The theorem has been further extended to magnetic systems for which both the electron density and spin density are fixed (“magnetic force theorem”) in calculating the magnetic exchange coupling constants of magnetic metals [4]. We shall also find that the theorem would hold even when the xc functional is nonlocal, if it is approximated to the second order of ${\vec{\rho }}_{S}$ as in Equation (22).

**Figure 1:**
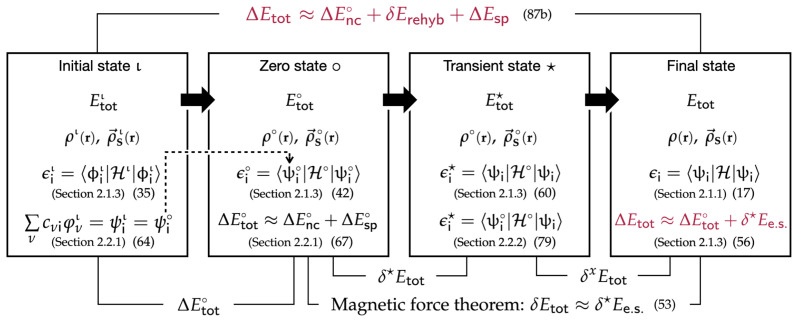
Magnetic states in various stages characterized by their electron densities, spin densities, eigenspinors, and eigenvalues, which are associated with the total electronic energy and its differences among the different magentic states. Relevant sections and equation numbers are given in parentheses. This work is based on the premise that $\rho^{\iota }\left(r\right)\approx \rho^{\circ }\left(r\right)\approx \rho \left(r\right)$.

Let us consider an initial state ι having the electron density $\rho^{\iota }$ and the spin density ${\vec{\rho }}_{S}^{\;\iota }$ (the leftmost panel in Figure 1). The Kohn–Sham Hamiltonian has the corresponding spin-dependent component $\nu^{S\iota }\cdot \vec{\sigma }$:

(33) $$\begin{array}{c} H^{\iota }=H^{\mathrm{nsp}}+\nu^{S\iota }\left(r\right)\cdot \vec{\sigma }\;. \end{array}$$

The eigenvalues and the spinors that are associated with the Hamiltonian are denoted as $\epsilon_{i}^{\iota }$ and $\phi_{i}^{\iota }$. The eigenspinor $\phi_{i}^{\iota }$ has a spatial component $\varphi_{i}^{\iota }$ and a unit spinor $\omega_{i}^{\iota }$ as a spin component with its spin orientation along $u_{i}^{\iota }$ (i.e., $\omega_{i}^{\iota †}\sigma \omega_{i}^{\iota }=u_{i}^{\iota }$):

(34) $$\begin{array}{ccc} \phi_{i}^{\iota } & = & \varphi_{i}^{\iota }\omega_{i}^{\iota }\;, \end{array}$$

and like $\epsilon_{i}$ in Equation (17), its energy is given as follows:

(35) $$\begin{array}{c} \epsilon_{i}^{\iota }=\langle \phi_{i}^{\iota }|H^{\iota }|\phi_{i}^{\iota }\rangle =\langle \phi_{i}^{\iota }|H^{\mathrm{nsp}}|\phi_{i}^{\iota }\rangle +\int \nu^{S\iota }\cdot {\vec{\rho }}_{Si}^{\;\iota }\;dr\;. \end{array}$$

For understanding of the magnetic force theorem, we examine a self-consistent procedure where the magnetic system first changes its spin density by $\delta {\vec{\rho }}_{S}^{\;\circ }$ from the initial spin density ${\vec{\rho }}_{S}^{\;\iota }$, so that the change (perturbation) leads to a new spin density denoted as “${\vec{\rho }}_{S}^{\;\circ }$” (the second panel fom the in Figure 1):

(36) $$\begin{array}{ccc} {\vec{\rho }}_{S}^{\;\circ } & = & {\vec{\rho }}_{S}^{\;\iota }+\delta {\vec{\rho }}_{S}^{\;\circ }\;. \end{array}$$

As illustrated in our previous work [17], an iterative SCF procedure starts with a new Kohn–Sham Hamiltonian having ${\vec{\rho }}_{S}^{\;\circ }$. Solving the Kohn–Sham equation with the new Hamiltonian provides new eigenfunctions and energies. The changes in the eigenfunctions induce an additional spin polarization (“induced spin density”), which is added to ${\vec{\rho }}_{S}^{\;\circ }$ to produce a new spin density for the next SCF cycle. This repeats until the final spin density $\rho_{s}$ and the total energy are obtained once the iterations reach self-consistent field (SCF) calculation results (the rightmost panel in Figure 1). Alternatively, the perturbational approach does not take this SCF step but provides and uses the analytical expressions of changes in the spinor energies/shapes and the approximate total energy change to understand the spin polarization effect in magnetic systems.

The starting magnetic state with ${\vec{\rho }}_{S}^{\;\circ }$ (called the “*zero state*” hereafter, denoted by “∘”) has the zeroth-order spinor $\psi_{i}^{\circ }$s:

(37) $$\begin{array}{ccc} \psi_{i}^{\circ } & = & \psi_{i}^{\circ }\omega_{i}^{\circ }\;, \end{array}$$

with their spin density vector ${\vec{\rho }}_{Si}^{\;\circ }$ that has its orbital density $\rho_{i}^{\circ }$ as its spatial part and its orientation along the unit vector $u_{i}^{\circ }$:

(38) $$\begin{array}{ccc} {\vec{\rho }}_{Si}^{\;\circ } & = & \psi_{i}^{\circ †}\vec{\sigma }\psi_{i}^{\circ }=\left(\psi_{i}^{\circ *}\psi_{i}^{\circ }\right)\left(\omega_{i}^{\circ †}\vec{\sigma }\omega_{i}^{\circ }\right)=\rho_{i}^{\circ }u_{i}^{\circ }\;. \end{array}$$

The zero-state spin density ${\vec{\rho }}_{S}^{\;\circ }$ is the sum of the spin densities of the occupied zeroth-order spinors:

(39) $$\begin{array}{ccc} {\vec{\rho }}_{S}^{\;\circ } & := & \sum_{i}\;n_{i}^{\circ }\psi_{i}^{\circ †}\vec{\sigma }\psi_{i}^{\circ }\;. \end{array}$$

From the viewpoint of perturbation theory, the spatial part ($\psi_{i}^{\circ }$) of the zeroth-order spinor $\psi_{i}^{\circ }$ is obtained via hybridization of the spatial parts ($\phi_{i}^{\iota }$’s) of $\phi_{i}^{\iota }$s, as discussed in Section 2.2.1.

Like in Equation (21), the total electronic energy difference between the initial state ι and the zero state ∘ ($\Delta E_{\mathrm{tot}}^{\circ }$ in Figure 1) is decomposed into the electronic structure energy component ($\Delta E_{e.s.}^{\circ }$) and the spin-dependent part of the double-count energy component ($\Delta E_{d.c.}^{S\circ }$):

(40) $$\begin{array}{ccc} \Delta E_{\mathrm{tot}}^{\circ } & := & E_{\mathrm{tot}}^{\circ }-E_{\mathrm{tot}}^{\iota }\approx \Delta E_{e.s.}^{\circ }+\Delta E_{d.c.}^{S\circ } \end{array}$$

where

(41a)ΔEe.s.∘:=Ee.s∘−Ee.sι=∑i(ni∘ϵi∘−niιϵiι),(41b)ΔEd.c.S∘:=Ed.c.S∘−Ed.c.Sι=−12∫(vS∘·ρ→S∘−νSι·ρ→Sι)dr.

Hereafter, the symbol ΔE denotes the change in an energy quantity with respect to the initial state ι as the reference state.

In Equation (41a), $\epsilon_{i}^{\circ }$ is the energy of the zeroth-order spinor, like $\epsilon_{i}$ in Equation (17):

(42) $$\begin{array}{ccc} \epsilon_{i}^{\circ } & := & \langle \psi_{i}^{\circ }|H^{\circ }|\psi_{i}^{\circ }\rangle =\langle \psi_{i}^{\circ }|H^{\mathrm{nsp}}|\psi_{i}^{\circ }\rangle +\int v^{S\circ }\cdot {\vec{\rho }}_{Si}^{\;\circ }\;dr\;, \end{array}$$

with the perturbed Hamiltonian,

(43) $$\begin{array}{ccc} H^{\circ } & = & H^{\mathrm{nsp}}+v^{S\circ }\cdot \vec{\sigma }\;, \end{array}$$

in which $v^{S\circ }$ is the spin-dependent part of the xc potential,

(44) $$\begin{array}{ccc} v^{S\circ } & := & -\frac{1}{2}\int {\vec{\rho }}_{S}^{\;\circ }\left(r^{\prime }\right)\cdot K_{\mathrm{xc}}\left(r,r^{\prime }\right)\;dr^{\prime }\overset{\mathrm{LSD}}{\approx }-\frac{1}{2}\kappa_{\mathrm{xc}}\;{\vec{\rho }}_{S}^{\;\circ }\;. \end{array}$$

The change in the spin-dependent part of the xc potential is then

(45) $$\begin{array}{ccc} \delta v^{S\circ } & := & v^{S\circ }-\nu^{S\iota }\approx -\frac{1}{2}\int \delta {\vec{\rho }}_{S}^{\;\circ }\left(r^{\prime }\right)\cdot K_{\mathrm{xc}}\left(r,r^{\prime }\right)\;dr^{\prime }\overset{\mathrm{LSD}}{\approx }-\frac{1}{2}\kappa_{\mathrm{xc}}\;\delta {\vec{\rho }}_{S}^{\;\circ }\;. \end{array}$$

In response to $\delta v^{S\circ }$ (or $\delta {\vec{\rho }}_{S}^{\;\circ }$) in the first step, the system relaxes to have the final spinors $\psi_{i}$ and the final spin density ${\vec{\rho }}_{S}$ (the far right panel in Figure 1). The additional change from ${\vec{\rho }}_{S}^{\;\circ }$ to ${\vec{\rho }}_{S}$ through the self-consistent relaxation procedure is the induced spin density ${\vec{\rho }}_{S}^{\;\mathrm{ind}}$ (${\vec{\rho }}_{S}^{\;\mathrm{ind}}:={\vec{\rho }}_{S}-{\vec{\rho }}_{S}^{\;\circ }$), which is fundamentally due to the change in the spinor wavefunctions during the relaxation ($\psi_{i}^{\circ }\to \psi_{i}$). Using Equation (15), the self-consistent Hamiltonian is expressed as follows:

(46) $$\begin{array}{ccc} H & = & H^{\mathrm{nsp}}+v^{S}\cdot \vec{\sigma }=H^{\circ }+v^{S\mathrm{ind}}\cdot \vec{\sigma }\;, \end{array}$$

with $v^{S}=v^{S\circ }+v^{S\mathrm{ind}}$, where the induced spin density (${\vec{\rho }}_{S}^{\;\mathrm{ind}}$) is given by the sum of the individual induced spin densities of the occupied spinors, which are defined as follows:

(47) $$\begin{array}{ccc} {\vec{\rho }}_{Si}^{\;\mathrm{ind}}\left(r\right) & := & \psi_{i}^{†}\vec{\sigma }\psi_{i}-\psi_{i}^{\circ †}\vec{\sigma }\psi_{i}^{\circ }\;. \end{array}$$

Like the approximate forms of $v^{S}$ and $v^{S\circ }$ in Equations (27) and (44),

(48) $$\begin{array}{ccc} v^{S\mathrm{ind}} & \approx & -\frac{1}{2}\int {\vec{\rho }}_{S}^{\;\mathrm{ind}}\left(r^{\prime }\right)\cdot K_{\mathrm{xc}}\left(r,r^{\prime }\right)\;dr^{\prime }\overset{\mathrm{LSD}}{\approx }-\frac{1}{2}\kappa_{\mathrm{xc}}\;{\vec{\rho }}_{S}^{\;\mathrm{ind}}\;. \end{array}$$

The magnetic force theorem concerns the change in the total electronic energy when the zero state ∘ relaxes to the final magnetic state (“$\delta E_{\mathrm{tot}}$” in Figure 1) [20]. To examine this energy change, the magnetic force theorem breaks down the self-consistent relaxation process into two sequential transitions of magnetic states. In the first transition, the system changes from the zero state ∘ to a fictitious transient state (denoted by “★”), shown in the second panel from the right in Figure 1. The transient state ★ keeps the spin density ${\vec{\rho }}_{S}^{\;\circ }$, but its eigenspinors are now $\psi_{i}$s: The eigenspinors of the final state. The second transition allows the transient state ★ to *relax* to the self-consistent solution of the final state (i.e., ${\vec{\rho }}_{S}^{\;\circ }\to {\vec{\rho }}_{S}$), while the eigenspinors do not change. Then, when we define the total energy changes during the first and second steps as $\delta^{★}E_{\mathrm{tot}}$ and $\delta^{x}E_{\mathrm{tot}}$, respectively:

(49) $$\begin{array}{ccc} \delta^{★}E_{\mathrm{tot}} & := & E_{\mathrm{tot}}^{★}-E_{\mathrm{tot}}^{\circ }\;,\;\delta^{x}E_{\mathrm{tot}}:=E_{\mathrm{tot}}-E_{\mathrm{tot}}^{★}\;, \end{array}$$

the overall total energy change from the zero state ∘ to the final state is given as their sum:

(50) $$\begin{array}{ccc} \delta E_{\mathrm{tot}} & := & E_{\mathrm{tot}}-E_{\mathrm{tot}}^{\circ }=\delta^{★}E_{\mathrm{tot}}+\delta^{x}E_{\mathrm{tot}}\;. \end{array}$$

We further decompose both $\delta^{★}E_{\mathrm{tot}}$ and $\delta^{x}E_{\mathrm{tot}}$ into their respective electronic structure energy and double-count energy components and eventually arrive at the following results:

(51a)δ★Etot=δ★Ee.s.+δ★Ed.c.≈δ★Ee.s.(51b)δxEtot=δxEe.s.+δxEd.c.≈12∫vSind·ρ→Sinddr.

The definition of $\delta^{★}E_{e.s.}$, the change in the electronic structure energy during the transition from the zero state ∘ to the transient state ★, is important:

(52) $$\begin{array}{ccc} \delta^{★}E_{e.s.} & := & E_{e.s.}^{★}-E_{e.s.}^{\circ }\;, \end{array}$$

because the summation of Equations (51a) and (51b) amounts to:

(53) $$\begin{array}{ccc} \delta E_{\mathrm{tot}} & \approx & \delta^{★}E_{e.s.}\;, \end{array}$$

which is correct *to the first order of* ${\vec{\rho }}_{S}^{\;\mathrm{ind}}$. This approximate relationship in Equation (53) is the magnetic force theorem without accounting for the effects of the change in volume of the system [20]). The explicit expressions for $\delta^{★}E_{e.s.}$ will be derived in Section 2.2.

Equation (51a) is due to the fact that the double-count energy is a function of only electron density and spin density and that there is no significant change in those during the first transition; hence, $\delta^{★}E_{d.c.}\approx 0$. Equation (51b) can be derived by first obtaining the following:

(54) $$\begin{array}{ccc} \delta^{x}E_{e.s.} & := & E_{e.s.}-E_{e.s.}^{★}=\sum_{i}\;n_{i}\langle \psi_{i}|H|\psi_{i}\rangle -\sum_{i}\;n_{i}\langle \psi_{i}|H^{\circ }|\rangle \psi_{i}=\sum_{i}\;n_{i}\langle \psi_{i}|\left(H-H^{\circ }\right)|\psi_{i}\rangle \\ & = & \int v^{S\mathrm{ind}}\cdot {\vec{\rho }}_{S}\;dr\approx \int v^{S}\cdot {\vec{\rho }}_{S}^{\;\mathrm{ind}}\;dr=\int v^{S\circ }\cdot {\vec{\rho }}_{S}^{\;\mathrm{ind}}\;dr+\int v^{S\mathrm{ind}}\cdot {\vec{\rho }}_{S}^{\;\mathrm{ind}}\;dr\;, \end{array}$$

by using the definition of the electronic structure energy as well as Equations (27), (46) and (48). Second, the approximate expression for $\delta^{x}E_{d.c.}$ in Equation (51b) is derived using Equations (27) and (48) as follows:

(55a)δxEd.c.:=Ed.c.−Ed.c.★≈δxEd.c.S≈−12∫vS·ρ→S−vS∘·ρ→S∘dr(55b)=−12∫vSind·ρ→S∘+vS∘·ρ→Sind+vSind·ρ→Sinddr(55c)≈−∫vS∘·ρ→Sinddr−12∫vSind·ρ→Sinddr.

Then, the summation of Equations (54) and (55c) gives Equation (51b), which leads to the magnetic force theorem.

The magnetic force theorem in Equation (53) allows us to determine the total electronic energy change from the initial magnetic state ι to the final state as follows:

(56) $$\begin{array}{ccc} \Delta E_{\mathrm{tot}} & := & E_{\mathrm{tot}}-E_{\mathrm{tot}}^{\iota }\approx \Delta E_{\mathrm{tot}}^{\circ }+\delta^{★}E_{e.s.}\;. \end{array}$$

By inserting $\Delta E_{\mathrm{tot}}$ into Equation (56), the magnetic exchange constant can be expressed as follows:

(57) $$\begin{array}{ccc} J_{\mathrm{ab}} & \approx & -\frac{1}{S_{a}S_{b}}{\left[\frac{\partial^{2}}{\partial \theta^{2}}\left(\Delta E_{\mathrm{tot}}^{\circ }+\delta^{★}E_{e.s.}\right)\right]}_{0}=-\frac{1}{S_{a}S_{b}}{\left[\frac{\partial^{2}}{\partial \theta^{2}}\left(E_{\mathrm{tot}}^{\circ }+\delta^{★}E_{e.s.}\right)\right]}_{0}\;. \end{array}$$

We may consider an alternative expression for $\Delta E_{\mathrm{tot}}$ in Equation (56). With the definition of $\Delta^{★}E_{e.s.}:=E_{e.s}^{★}-E_{e.s}^{\iota }$, analogous to that of $E_{e.s}^{\circ }$ in Equation (41a), the combination of Equations (40), (52) and (56) leads to the following:

(58) $$\begin{array}{ccc} \Delta E_{\mathrm{tot}} & \approx & \Delta^{★}E_{e.s.}+\Delta E_{d.c.}^{S\circ }\;. \end{array}$$

The energy terms in Equation (58) can be rearranged so that the total energy change is given as the changes in the spin-dependent energy terms from the initial state to the transient state:

(59) $$\begin{array}{ccc} \Delta E_{\mathrm{tot}} & \approx & \left(\sum_{i}n_{i}\epsilon_{i}^{★}-\frac{1}{2}\int v^{S\circ }\cdot {\vec{\rho }}_{S}^{\;\circ }\;dr\right)-\left(\sum_{i}n_{i}^{\iota }\epsilon_{i}^{\iota }-\frac{1}{2}\int \nu^{S\iota }\cdot {\vec{\rho }}_{S}^{\;\iota }\;dr\right)\;. \end{array}$$

It is noted that in calculating $\Delta E_{\mathrm{tot}}$ in Equation (59), the only term that needs to be determined is the spinor energy of the transient state ★ for which $H^{\circ }$, not H, is employed in its expression:

(60) $$\begin{array}{ccc} \epsilon_{i}^{★} & := & \langle \psi_{i}|H^{\circ }|\psi_{i}\rangle \;. \end{array}$$

Finally, by using the magnetic force theorem in the form of Equation (59), $J_{\mathrm{ab}}$ in Equation (2) is rewritten as follows:

(61) $$\begin{array}{cc} J_{\mathrm{ab}} & \approx -\frac{1}{S_{a}S_{b}}{\left[\frac{\partial^{2}}{\partial \theta^{2}}\left(\sum_{i}n_{i}\epsilon_{i}^{★}-\frac{1}{2}\int v^{S\circ }\cdot {\vec{\rho }}_{S}^{\;\circ }\;dr\right)\right]}_{0}\;, \end{array}$$

after dropping the constant energy terms of the initial state. In other words, the magnetic exchange constant is the second-order change in the electronic structure energy of the transient state ★, which is corrected by the double-count energy of the zero state ∘.

### 2.2. Perturbation Theory with Spinors

Estimation of the transient state spinor energy $\epsilon_{i}^{★}$ in Equation (60) or $\delta^{★}E_{e.s.}$ in Equation (57) can be performed using a perturbational approach where we derive explicit mathematical expressions for the perturbed spinors and their energies. We will do so in the following two subsections by applying the Brillioun–Wigner perturbation method and Green’s function perturbation method. For this purpose, we follow Epstein–Nesbet partitioning by decomposing $H^{\circ }$ with a fictitious Epstein–Nesbet zeroth-order Hamiltonian $H^{⊖}$ and the perturbation $H^{\prime }$:

(62) $$\begin{array}{c} H^{\circ }=H^{⊖}+H^{\prime }\;, \end{array}$$

so that they provide a *diagonal* matrix $H^{⊖}$ and a *hollow* matrix $H^{\prime }$, respectively, with the basis set $\left\{\psi_{i}^{\circ }\right\}$:

(63a)H⊖ji:=ϵi∘δji,(63b)Hji′:=〈ψj∘|H∘|ψi∘〉[j≠i],

where $\delta_{ji}$ and [] are a Kronecker delta and an Iverson bracket, respectively [16,19].

In this scheme, the Epstein–Nesbet partitioning identifies the zeroth-order spinors, $\psi_{i}^{\circ }$, as the eigenspinors of the zeroth-state ∘ (the second panel from the left in Figure 1) hose energies are given by the diagonal matrix $H^{⊖}$ in its zero state. Upon the perturbation given by the off-diagonal Hamilton matrix elements, $H_{ji}^{\prime }$, the $\psi_{i}^{\circ }$s interact (mix) to form the eigenspinors, $\psi_{i}$, of the transient state ★ (the second panel from the right in Figure 1). The perturbational analysis in the rest of this section provides the explicit expressions of $\psi_{i}^{\circ }$s as the perturbed spinors and of their energies, $\epsilon_{i}^{★}$s. This further allows us to obtain the explicit expressions for $\delta^{★}E_{e.s.}$, which leads to the calculation of the magnetic exchange coupling constant via Equation (57) or (61). These expressions can be rewritten in terms of the spin polarization energy, as shown later in Equation (73).

#### 2.2.1. Choice of the Zeroth-Order Spinors and the Zero-State Spin Polarization Energy

Before we proceed, the proper choice of the zeroth-order spinors requires understanding of the desired nature of the zeroth-order spinors. For localized electrons, the spatial component of the zeroth-order spinors are *magnetic orbitals*, and they are used to describe both spin-independent and spin-dependent interactions of the magnetically ”active” electrons. For ”passive” electrons, the original delocalized orbitals are used without modification. We can find the magnetic orbitals through various localization procedures as a linear combination of the *spatial part* of the eigenfunctions of the initial (unperturbed) state (the reference state “ι”) [16,19]. In practice, the ferromagnetic state of the magnetic system may be chosen as a preferred reference state, because the spinors in the ferromagnetic state have the same spin direction and thus are most delocalized in comparison to those in other magnetic states.

The resulting magnetic orbitals are the same for all different magnetic states:

(64) $$\begin{array}{ccc} \psi_{i}^{\circ } & = & \psi_{i}^{\iota }=\sum_{\nu }\;c_{\nu i}\varphi_{\nu }^{\iota }\;, \end{array}$$

and the corresponding *magnetic spinors*, the zeroth-order spinors, are obtained by multiplying by the magnetic orbitals’ appropriate unit spinors: $\omega_{i}^{\circ }$ in $\psi_{i}^{\circ }$ (Equation (37) and $\omega_{i}^{\iota }$ in $\psi_{i}^{\iota }=\psi_{i}^{\iota }\omega_{i}^{\iota }$. In other words, the magnetic orbitals are chosen to reproduce both ${\vec{\rho }}_{S}^{\;\iota }$ and ${\vec{\rho }}_{S}^{\;\circ }$ after combining the corresponding unit spinors while not changing the total electron density:

(65a)∑ini∘ψiι†σ→ψiι=∑ini∘|ψi∘|2uiι≈ρ→Sι,∑ini∘ψiι†ψiι≈ρι(65b)∑ini∘ψi∘†σ→ψi∘=∑ini∘|ψi∘|2ui∘=ρ→S∘,∑ini∘ψi∘†ψi∘=ρ∘≈ρι.

We note that the magnetic orbitals are generally noncanonical, with non-zero orbital interactions among them:

(66) $$\begin{array}{c} \varepsilon_{ji}^{\circ }:=\langle \psi_{j}^{\circ }|H^{\mathrm{nsp}}|\psi_{i}^{\circ }\rangle \;. \end{array}$$

Since the Hamiltonian $H^{\mathrm{nsp}}$ is spin-independent, the expression for $\varepsilon_{ji}^{\circ }$ in Equation (66) connects at least some energy components of the magnetic exchange interactions to the *orbital* interactions of the magnetic spinors, as described in Section 2.3.1.

We examine the change in the total electronic energy from the initial state to the zero state ($\Delta E_{\mathrm{tot}}^{\circ }$) (the second panel from the left in Figure 1). Since there is no induced spin polarization in this transition, from Equation (31) we find

(67) $$\begin{array}{ccc} \Delta E_{\mathrm{tot}}^{\circ } & \approx & \Delta E_{\mathrm{tot}}^{S}\approx \Delta E_{\mathrm{nc}}^{\circ }+\Delta E_{\mathrm{sp}}^{\circ }\;, \end{array}$$

where we define

(68a)ΔEnc∘:=∑ini∘εii∘−niι〈ϕiι|Hnsp|ϕiι〉,(68b)ΔEsp∘:=Esp∘−Espι=−12∫(vS∘·ρ→S∘−νSι·ρ→Sι)dr.

$E_{\mathrm{sp}}^{\circ }$ is the spin polarization energy of the zero state ∘:

(69) $$\begin{array}{ccc} E_{\mathrm{sp}}^{\circ } & \approx & \frac{1}{2}\int v^{S\circ }\cdot {\vec{\rho }}_{S}^{\;\circ }\;dr\overset{\mathrm{LSD}}{\approx }-\frac{1}{4}\int \kappa_{\mathrm{xc}}\;{\left(\rho_{S}^{\circ }\right)}^{2}\;dr\;, \end{array}$$

while $E_{\mathrm{sp}}^{\;\iota }$ is that of the initial state ι:

(70) $$\begin{array}{ccc} E_{\mathrm{sp}}^{\iota } & \approx & \frac{1}{2}\int \nu^{S\iota }\cdot {\vec{\rho }}_{S}^{\;\iota }\;dr\overset{\mathrm{LSD}}{\approx }-\frac{1}{4}\int \kappa_{\mathrm{xc}}\;{\left(\rho_{S}^{\;\iota }\right)}^{2}\;dr\;. \end{array}$$

Therefore, from Equations (56) and (67), we have the following:

(71) $$\begin{array}{ccc} \Delta E_{\mathrm{tot}} & \approx & \Delta E_{\mathrm{nc}}^{\circ }+\Delta E_{\mathrm{sp}}^{\circ }+\delta^{★}E_{e.s.}\;. \end{array}$$

Since different magnetic states share the same magnetic orbitals to start with, the $\varepsilon_{ji}^{\circ }$ values are the same for the *active* electrons among all the magnetic states, including the initial state. Meanwhile, the zeroth-order spinors of the *passive* electrons would keep their original shape and spin direction, and the $\varepsilon_{ji}^{\circ }$s remain unchanged by the perturbation. An important implication of these observations is that $\Delta E_{\mathrm{nc}}^{\circ }$ in Equations (67) and (71) is *a constant*, including 0, between any two magnetic states:

(72) $$\begin{array}{ccc} \Delta E_{\mathrm{nc}}^{\circ } & \approx & a\mathrm{constant}\;. \end{array}$$

Therefore, in Equation (57), the magnetic exchange constant $J_{\mathrm{ab}}$ can be re-expressed by dropping $\Delta E_{\mathrm{nc}}^{\circ }$ and using only the spin polarization energy change or spin polarization energy itself of the zero state, in place of the total electronic energy of the zero state:

(73) $$\begin{array}{ccc} J_{\mathrm{ab}} & \approx & -\frac{1}{S_{a}S_{b}}{\left[\frac{\partial^{2}}{\partial \theta^{2}}\left(\Delta E_{\mathrm{sp}}^{\circ }+\delta^{★}E_{e.s.}\right)\right]}_{0}=-\frac{1}{S_{a}S_{b}}{\left[\frac{\partial^{2}}{\partial \theta^{2}}\left(E_{\mathrm{sp}}^{\circ }+\delta^{★}E_{e.s.}\right)\right]}_{0}\;. \end{array}$$

Henceforward, we may consider this expression of $J_{\mathrm{ab}}$ in Equation (73) to be most convenient, as can be seen in Section 2.3. The explicit expressions for $\delta^{★}E_{e.s.}$ are derived in Section 2.2.3 and Section 2.2.4 for general cases, as well as at the end of Section 2.2.2 for the special case where only passive electrons are responsible for magnetic exchange interactions.

#### 2.2.2. Interaction Energy Terms of the Zeroth-Order Spinors

After choosing the zeroth-order spinors, $\psi_{i}^{\circ }$, we express $\psi_{i}$ as the perturbed spinors originating from them by adding a small perturbation component $\delta \psi_{i}^{\circ }$ that needs to be determined via the perturbation theory:

(74) $$\begin{array}{c} \psi_{i}=\psi_{i}^{\circ }+\delta \psi_{i}^{\circ }=\psi_{i}^{\circ }+\sum_{j\neq i}t_{ji}\psi_{j}^{\circ }\;, \end{array}$$

where $t_{ii}=1$ and $\sum_{j\neq i}t_{ji}=0$ under the intermediate normalization condition with a parallel gauge transformation:

(75) $$\begin{array}{c} \langle \psi_{i}^{\circ }|\psi_{i}\rangle =\langle \psi_{i}|\psi_{i}^{\circ }\rangle =1\;. \end{array}$$

The spinors that satisfy this normalization condition are accurate to the first order of the spin density vector change, which is consistent with the magnetic force theorem. The spin density vector ${\vec{\rho }}_{Si}$ of the perturbed spinor $\psi_{i}$ is given as follows:

(76) $$\begin{array}{ccc} {\vec{\rho }}_{Si} & := & {\vec{\rho }}_{Si}^{\;\circ }+{\vec{\rho }}_{Si}^{\;\mathrm{ind}}\;, \end{array}$$

when from Equation (47), we define the intermediate induced spin density vector ${\vec{\rho }}_{Si}^{\;\mathrm{ind}}$ as follows:

(77) $$\begin{array}{ccc} {\vec{\rho }}_{Si}^{\;\mathrm{ind}}:=\psi_{i}^{\circ †}\vec{\sigma }\;\delta \psi_{i} & = & \sum_{j\neq i}\left(\psi_{i}^{\circ †}\vec{\sigma }\psi_{j}^{\circ }\right)\;t_{ji} \end{array}\;.$$

Then, the total intermediate spin density vector, ${\vec{\rho }}_{S}$, and the total intermediate induced spin density vector, ${\vec{\rho }}_{S}^{\;\mathrm{ind}}$, can be obtained as the summations of ${\vec{\rho }}_{Si}$ and ${\vec{\rho }}_{Si}^{\;\mathrm{ind}}$, respectively, over all the occupied spinors:

(78) $$\begin{array}{ccc} {\vec{\rho }}_{S} & := & \sum_{i}\;n_{i}{\vec{\rho }}_{Si}=\sum_{i}\;n_{i}\left({\vec{\rho }}_{Si}^{\;\circ }+{\vec{\rho }}_{Si}^{\;\mathrm{ind}}\right)={\vec{\rho }}_{S}^{\;\circ }+{\vec{\rho }}_{S}^{\;\mathrm{ind}}\;,\;\mathrm{where}\;\;{\vec{\rho }}_{S}^{\;\mathrm{ind}}:=\sum_{i}\;n_{i}{\vec{\rho }}_{Si}^{\;\mathrm{ind}}\;. \end{array}$$

Henceforth, any suitable choice of the zeroth-order spinors should lead to an ${\vec{\rho }}_{S}$ value that is reasonably close to ${\vec{\rho }}_{S}$(i.e., ${\vec{\rho }}_{Si}^{\;\mathrm{ind}}\approx {\vec{\rho }}_{Si}^{\;\mathrm{ind}}$). Equation (77) will be employed hereafter in place of Equation (47). Under the same normalization condition, the transient state spinor energy is rewritten as follows:

(79) $$\begin{array}{ccc} \epsilon_{i}^{★} & = & \langle \psi_{i}^{\circ }|H^{\circ }|\psi_{i}\rangle =\epsilon_{i}^{\circ }+\sum_{j\neq i}H_{ij}^{\prime }\;t_{ji}\;, \end{array}$$

with $\epsilon_{i}^{\circ }$ as the zero-state spinor energy and the rest as the spinor energy change due to the perturbation, employing Equations (63) and (74).

In the absence of the spin–orbit interactions, the matrix elements in Equation (63) can be discomposed into two components by separating the Hamiltonian given in Equation (43) to the $v^{S\circ }$-independent and -dependent terms:

(80a)ϵi∘=εii∘+Δ˜iiS∘,(80b)Hji′=ϵji∘+Δ˜jiS∘,

where $\epsilon_{ji}^{\circ }$ is the interaction energy *via*$H^{\mathrm{nsp}}$ between the magnetic spinors with different spin orientations:

(81) $$\begin{array}{ccc} \epsilon_{ji}^{\circ } & := & \langle \psi_{j}^{\circ }|H^{\mathrm{nsp}}|\psi_{i}^{\circ }\rangle =\varepsilon_{ji}^{\circ }\left(\omega_{j}^{\circ †}\omega_{i}^{\circ }\right)\;. \end{array}$$

The last identity applies when the $\omega_{i}^{\circ }$s are r-independent, as we would normally expect for magnetic spinors. Meanwhile, ${\tilde{\Delta }}_{ii}^{S\circ }$ and ${\tilde{\Delta }}_{ji}^{S\circ }$ are defined as follows:

(82a)Δ˜iiS∘:=∫vS∘·ρ→Si∘dr≈−12∫∫ρ→S∘(r′)·Kxc(r,r′)·ρ→Si∘(r)dr′dr≈LSD−12∫κxcρ→S∘·ρ→Si∘dr,(82b)Δ˜jiS∘:=∫vS∘·ρ→Sji∘dr≈−12∫∫ρ→S∘(r′)·Kxc(r,r′)·ρ→Sji∘(r)dr′dr≈LSD−12∫κxcρ→S∘·ρ→Sji∘dr.

In Equation (82b), the ${\vec{\rho }}_{Sji}^{\;\circ }$ is the generalization of ${\vec{\rho }}_{Si}^{\;\circ }$ in Equation (38):

(83) $$\begin{array}{ccc} {\vec{\rho }}_{Sji}^{\;\circ } & := & \psi_{j}^{\circ †}\vec{\sigma }\psi_{i}^{\circ }=\left(\psi_{j}^{\circ *}\psi_{i}^{\circ }\right)\left(\omega_{j}^{\circ †}\vec{\sigma }\omega_{i}^{\circ }\right)\;. \end{array}$$

We note that in collinear magnetism, the off-diagonal terms given in Equations (81) and (82b) become zero between the spinors with opposite spin directions (↑ and ↓).

Insertion of Equation (80b) in Equation (79) and application of Equation (77) leads to the energy of the spinors in the transient state ★:

(84) $$\begin{array}{ccc} \epsilon_{i}^{★} & = & \epsilon_{i}^{\circ }+\sum_{j\neq i}\epsilon_{ij}^{\circ }\;t_{ji}+\int v^{S\circ }\cdot {\vec{\rho }}_{Si}^{\;\mathrm{ind}}\;dr\;, \end{array}$$

and consequently,

(85) $$\begin{array}{ccc} \delta^{★}E_{e.s.} & \approx & \delta E_{\mathrm{rehyb}}+\int v^{S\circ }\cdot {\vec{\rho }}_{S}^{\;\mathrm{ind}}\;dr\overset{\mathrm{LSD}}{\approx }\delta E_{\mathrm{rehyb}}-\frac{1}{2}\int \kappa_{\mathrm{xc}}\;{\vec{\rho }}_{S}^{\;\circ }\cdot {\vec{\rho }}_{S}^{\;\mathrm{ind}}\;dr\;, \end{array}$$

where $\delta E_{\mathrm{rehyb}}$ is the energy lowering by the mixing (or rehybridization/delocalization) of the magnetic spinors *via*$H^{\mathrm{nsp}}$, although this distinction may not be unambiguous when ${\tilde{\Delta }}_{ji}^{S\circ }\neq 0$:

(86) $$\begin{array}{ccc} \delta E_{\mathrm{rehyb}} & := & \sum_{i}n_{i}\sum_{j\neq i}\epsilon_{ij}^{\circ }\;t_{ji}=\sum_{i}n_{i}\sum_{j\neq i}\langle \psi_{i}^{\circ }|H^{\mathrm{nsp}}|\psi_{j}^{\circ }\rangle t_{ji}\;. \end{array}$$

After inserting Equation (85) into Equation (71), $\Delta E_{\mathrm{tot}}$ is re-expressed as follows:

(87a)ΔEtot≈ΔEnc∘+δErehyb+ΔEsp∘+∫vS∘·ρ→Sinddr(87b)≈ΔEnc∘+δErehyb+ΔEsp,

where $\Delta E_{\mathrm{sp}}$ is the change in the spin polarization energy from the initial state to the final state. The spin polarization energy of the final state $E_{\mathrm{sp}}$ is given in an approximate form:

(88) $$\begin{array}{ccc} E_{\mathrm{sp}} & \approx & \frac{1}{2}\int v^{S\circ }\cdot \left({\vec{\rho }}_{S}^{\;\circ }+2{\vec{\rho }}_{S}^{\;\mathrm{ind}}\right)\;dr\overset{\mathrm{LSD}}{\approx }-\frac{1}{4}\int \kappa_{\mathrm{xc}}\;{\vec{\rho }}_{S}^{\;\circ }\cdot \left({\vec{\rho }}_{S}^{\;\circ }+2{\vec{\rho }}_{S}^{\;\mathrm{ind}}\right)\;dr\;. \end{array}$$

This expression is different from the one in Equation (30) in the second order of ${\vec{\rho }}_{S}^{\;\mathrm{ind}}$, which is consistent with the approximation in the magnetic force theorem (Equation (53). We further note that the expression $\Delta E_{\mathrm{tot}}$ of Equation (87b) is equivalent to what we can obtain from the change in $E_{\mathrm{tot}}^{S}$ (Equation (31)), in the framework of the perturbational approach.

In case the zeroth-order spinors are *canonical with* $H^{\mathrm{nsp}}$ (i.e., $\varepsilon_{ji}^{\circ }=0$, and thus $\epsilon_{ji}^{\circ }=0$ for j≠i in Equation (81), $\Delta E_{\mathrm{nc}}^{\circ }=0$; $\delta E_{\mathrm{rehyb}}=0$; thus,

(89) $$\begin{array}{ccc} \Delta E_{\mathrm{tot}} & \approx & \Delta E_{\mathrm{sp}}\;. \end{array}$$

Likewise, the energy of the canonical spinors does not have the energy component associated with the rehybridization:

(90) $$\begin{array}{ccc} \epsilon_{i}^{★} & \approx & \langle \psi_{i}^{\circ }|H^{\circ }|\psi_{i}\rangle =\epsilon_{i}^{\circ }+\int v^{S\circ }\cdot {\vec{\rho }}_{Si}^{\;\mathrm{ind}}\;dr\;, \end{array}$$

nor does $\delta^{★}E_{e.s.}$:

(91) $$\begin{array}{ccc} \delta^{★}E_{e.s.} & \approx & \int v^{S\circ }\cdot {\vec{\rho }}_{S}^{\;\mathrm{ind}}\;dr\overset{\mathrm{LSD}}{\approx }-\frac{1}{2}\int \kappa_{\mathrm{xc}}\;{\vec{\rho }}_{S}^{\;\circ }\cdot {\vec{\rho }}_{S}^{\;\mathrm{ind}}\;dr\;. \end{array}$$

#### 2.2.3. Brillouin-Wigner Perturbation Method

In applying Brillioun–Wigner perturbation theory with the matrix elements in Equation (63) of the Hamiltonian of Equation (62), we would solve the following equation self-consistently in principle [21,22]:

(92) $$\begin{array}{cc} \; & \left(\epsilon_{i}-H^{⊖}\right)|\psi_{i}\rangle =H^{\prime }|\psi_{i}\rangle \;, \end{array}$$

by first constructing the projection operator with the zeroth-order spinors ($\psi_{i}^{\circ }$s) as the basis functions of a complete set:

(93) $$\begin{array}{cc} Q & :=\sum_{j\neq i}|\psi_{j}^{\circ }\rangle \langle \psi_{j}^{\circ }|=\sigma_{0}-|\psi_{i}^{\circ }\rangle \langle \psi_{i}^{\circ }|\;, \end{array}$$

and its resolvent:

(94) $$\begin{array}{cc} R^{\circ } & :={\left(\epsilon_{i}-H^{⊖}\right)}^{-1}Q=Q{\left(\epsilon_{i}-H^{⊖}\right)}^{-1}\;. \end{array}$$

The projection operator and resolvent are convenient to express the change in the spinors due to perturbation:

(95) $$\begin{array}{cc} |\delta \psi_{i}\rangle := & |\psi_{i}\rangle -|\psi_{i}^{\circ }\rangle =Q|\psi_{i}\rangle =R^{\circ }H^{\prime }|\psi_{i}\rangle \;. \end{array}$$

This leads to a series expansion of $\psi_{i}$ (Dyson equation for wavefunctions):

(96) $$\begin{array}{cc} |\psi_{i}\rangle & =|\psi_{i}^{\circ }\rangle +R^{\circ }H^{\prime }|\psi_{i}\rangle =|\psi_{i}^{\circ }\rangle +R^{\circ }H^{\prime }|\psi_{i}^{\circ }\rangle +{\left(R^{\circ }H^{\prime }\right)}^{2}|\psi_{i}\rangle \\ \; & =\sum_{n=0}^{\infty }{\left(R^{\circ }H^{\prime }\right)}^{n}|\psi_{i}^{\circ }\rangle \;, \end{array}$$

and by using the spectral representation of $R^{\circ }$:

(97) $$\begin{array}{c} R^{\circ }=\sum_{j\neq i}\frac{\left|\psi_{j}^{\circ }\rangle \langle \psi_{j}^{\circ }\right|}{\epsilon_{i}-\epsilon_{j}^{\circ }}\;, \end{array}$$

the approximate expression for $|\psi_{i}\rangle$ is given as follows:

(98) $$\begin{array}{cc} |\psi_{i}\rangle \; & =|\psi_{i}^{\circ }\rangle +\sum_{j\neq i}\frac{|\psi_{j}^{\circ }\rangle H_{ji}^{\prime }}{\epsilon_{i}-\epsilon_{j}^{\circ }}+\sum_{j\neq i}\sum_{k\neq i}\frac{|\psi_{j}^{\circ }\rangle H_{jk}^{\prime }H_{ki}^{\prime }}{\left(\epsilon_{i}-\epsilon_{j}^{\circ }\right)\left(\epsilon_{i}-\epsilon_{k}^{\circ }\right)}+...\;. \end{array}$$

It is noted that $R^{\circ }$ has the same mathematical form as Green’s function, except that the former is defined only for each eigenfunction and eigenvalue, while the latter is in principle continuously in energy [23].

In Equation (98), the perturbed energy $\epsilon_{i}$ is still unknown and needs to be determined through iterations. Alternatively, one can substitute $\epsilon_{i}$ on the right side of the equation with $\epsilon_{i}^{\circ }$ Therefore, Equation (98) is re-expressed in an approximation as follows:

(99) $$\begin{array}{c} |\psi_{i}\rangle =|\psi_{i}^{\circ }\rangle +\sum_{j\neq i}\frac{|\psi_{j}^{\circ }\rangle H_{ji}^{\prime }}{\epsilon_{i}^{\circ }-\epsilon_{j}^{\circ }}+\sum_{j\neq i}\sum_{k\neq i}\frac{|\psi_{j}^{\circ }\rangle H_{jk}^{\prime }H_{ki}^{\prime }}{\left(\epsilon_{i}^{\circ }-\epsilon_{j}^{\circ }\right)\left(\epsilon_{i}^{\circ }-\epsilon_{k}^{\circ }\right)}+...\;, \end{array}$$

and insertion of Equation (99) into Equation (79) gives the expression for the transient state spinor energy:

(100a)ϵi★=ϵi∘+∑j≠iHij′Hji′ϵi∘−ϵj∘+∑k≠iHjk′Hki′(ϵi∘−ϵj∘)(ϵi∘−ϵk∘)+...(100b)=ϵi∘+∑j≠iHij′Hji′ϵi∘−ϵj∘+∑j≠i∑k≠iHij′Hjk′Hki′(ϵi∘−ϵj∘)(ϵi∘−ϵk∘)+....

By comparing Equation (99) with Equation (74), we find spinor coefficients, $t_{ii}=1$, and for j≠i,

(101) $$\begin{array}{c} t_{ji}=\frac{H_{ji}^{\prime }}{\epsilon_{i}^{\circ }-\epsilon_{j}^{\circ }}+\sum_{k\neq i}\frac{H_{jk}^{\prime }H_{ki}^{\prime }}{\left(\epsilon_{i}^{\circ }-\epsilon_{j}^{\circ }\right)\left(\epsilon_{i}^{\circ }-\epsilon_{k}^{\circ }\right)}+...\;. \end{array}$$

After multiplying $n_{i}^{\circ }$ by $\epsilon_{i}^{★}$ and summing them over all the spinors, we obtain the following:

(102) $$\begin{array}{ccc} E_{e.s.}^{★} & = & \sum_{i}n_{i}^{\circ }\epsilon_{i}^{\circ }+\sum_{i}\;n_{i}^{\circ }\left[\sum_{j\neq i}\frac{H_{ij}^{\prime }H_{ji}^{\prime }}{\epsilon_{i}^{\circ }-\epsilon_{j}^{\circ }}+\sum_{j\neq i}\sum_{k\neq i}\frac{H_{ij}^{\prime }H_{jk}^{\prime }H_{ki}^{\prime }}{\left(\epsilon_{i}^{\circ }-\epsilon_{j}^{\circ }\right)\left(\epsilon_{i}^{\circ }-\epsilon_{k}^{\circ }\right)}+...\right]\;, \end{array}$$

and thus,

(103) $$\begin{array}{ccc} \delta^{★}E_{e.s.} & = & \sum_{i}\;n_{i}^{\circ }\left[\sum_{j\neq i}\frac{H_{ij}^{\prime }H_{ji}^{\prime }}{\epsilon_{i}^{\circ }-\epsilon_{j}^{\circ }}+\sum_{j\neq i}\sum_{k\neq i}\frac{H_{ij}^{\prime }H_{jk}^{\prime }H_{ki}^{\prime }}{\left(\epsilon_{i}^{\circ }-\epsilon_{j}^{\circ }\right)\left(\epsilon_{i}^{\circ }-\epsilon_{k}^{\circ }\right)}+...\right]\;. \end{array}$$

It is important to note that in the double summation in Equations (102) and (103), the interactions between occupied spinors cancel each other out and thus the *net* interactions come only from between *occupied* spinors and *unoccupied* spinors.

#### 2.2.4. Green’s Function Perturbation Method for Continuous Energy Systems

For the systems with a continuous energy spectrum, Green’s function perturbation method has been extensively used to calculate the magnetic exchange interaction energy in the form of the Lichtenstein formula [4]. In this section, we show that the results from the method are equivalent to the ones from the Brillouin–Wigner perturbation method used in the previous section. From the result of the magnetic force theorem given in Equation (56), the total electronic energy change due to the perturbation is given as follows:

(104) $$\begin{array}{ccc} \Delta E_{\mathrm{tot}} & \approx & \Delta E_{\mathrm{tot}}^{\circ }+\delta^{★}E_{b.s.}\approx \Delta E_{\mathrm{nc}}^{\circ }+\Delta E_{\mathrm{sp}}^{\circ }+\delta^{★}E_{b.s.}\;, \end{array}$$

where $\delta^{★}E_{b.s.}$ is the additional band structure energy change due to the perturbation. Analogous to Equation (73), the expression for the magnetic exchange constant $J_{\mathrm{ab}}$ is given as follows:

(105) $$\begin{array}{ccc} J_{\mathrm{ab}} & \approx & -\frac{1}{S_{a}S_{b}}{\left[\frac{\partial^{2}}{\partial \theta^{2}}\left(\Delta E_{\mathrm{sp}}^{\circ }+\delta^{★}E_{b.s.}\right)\right]}_{0}=-\frac{1}{S_{a}S_{b}}{\left[\frac{\partial^{2}}{\partial \theta^{2}}\left(E_{\mathrm{sp}}^{\circ }+\delta^{★}E_{b.s.}\right)\right]}_{0}\;. \end{array}$$

In the calculation of $\delta^{★}E_{b.s.}$ in Equation (105), we begin with the general expression for the electronic structure energy of the continuous energy systems as follows:

(106) $$\begin{array}{cc} E_{b.s.}\; & =\sum_{i}^{occ.}\epsilon_{i}=\int_{-\infty }^{\epsilon_{F}}d\epsilon \;\epsilon \;n\left(\epsilon \right)=\int_{-\infty }^{\epsilon_{F}}d\epsilon \;\epsilon \frac{dN(\epsilon )}{d\epsilon }={\left[\epsilon N(\epsilon )\right]}_{-\infty }^{\epsilon_{F}}-\int_{-\infty }^{\epsilon_{F}}d\epsilon \;N\left(\epsilon \right)\\ \; & =\epsilon_{F}N\left(\epsilon_{F}\right)-\int_{-\infty }^{\epsilon_{F}}d\epsilon \;N\left(\epsilon \right)\;, \end{array}$$

where n(ϵ) is the density of states and N(ϵ) is the integrated density of the following states:

(107) $$\begin{array}{c} N\left(\epsilon \right)=\int_{-\infty }^{\epsilon }d\epsilon \;n\left(\epsilon \right)\;. \end{array}$$

It is noted that both n(ϵ) and N(ϵ) functions depend on the band structure of the system, while $N(\epsilon_{F})$ is the same for perturbed and unperturbed systems because the total number of electrons ($N_{\mathrm{tot}}$) does not change after the perturbation: $N_{\mathrm{tot}}=N^{\iota }\left(\epsilon_{F}^{\iota }\right)=N^{\circ }\left(\epsilon_{F}^{\circ }\right)=N^{★}\left(\epsilon_{F}^{★}\right)$.

Then, $\delta^{★}E_{b.s.}$ can be simplified after using the expression for the band structure energy in Equation (106) for both $E_{b.s.}^{★}$ and $E_{b.s.}^{\circ }$, subtracting the latter from the former:

(108a)δ★Eb.s.:=Eb.s.★−Eb.s.∘=(ϵF★−ϵF∘)Ntot−∫−∞ϵF★dϵN★(ϵ)−∫−∞ϵF∘dϵN∘(ϵ)(108b)≈−∫−∞ϵF∘dϵΔ★N(ϵ),

where $\Delta^{★}N\left(\epsilon \right)$ is defined as the change in the integrated density of state due to the interactions of the spinors:

(109) $$\begin{array}{ccc} \Delta^{★}N\left(\epsilon \right) & := & N^{★}\left(\epsilon \right)-N^{\circ }\left(\epsilon \right)\;. \end{array}$$

The expression in Equation (108b) is precise, as long as the occupation numbers do not change, which is what we expect, at least for discrete electronic energy systems. Otherwise, the approximation in Equation (108b) holds when $\epsilon_{F}^{★}\approx \epsilon_{F}^{\circ }$, or when

(110) $$\begin{array}{ccc} n^{★}\left(\epsilon_{F}^{★}\right) & \approx & -\frac{2}{3}{\left[\frac{dn^{★}\left(\epsilon \right)}{d\epsilon }\right]}_{\epsilon_{F}^{★}}\left(\epsilon_{F}^{★}-\epsilon_{F}^{\circ }\right)\;, \end{array}$$

to the the second order of $N^{★}\left(\epsilon \right)$.

What is left is to find the expression for the energy integral of $\Delta^{★}N\left(\epsilon \right)$ in Equation (108b) using Green’s function perturbation method. Within the Epstein–Nesbet partitioning scheme, the time-independent Green’s functions for the unperturbed and perturbed electronic systems (*g* and *G*) satisfy the following inhomogeneous equations:

(111) $$\begin{array}{c} (z-H^{⊖})g\left(z\right)=1\;,\;\left(z-H\right)G\left(z\right)=1\;. \end{array}$$

G(z) and g(z) are related through the T-matrix operator (also known as the ”scattering path operator”):

(112) $$\begin{array}{cc} G & =g+gH^{\prime }G=g+GH^{\prime }g=g+gTg\;, \end{array}$$

from which we note

(113) $$\begin{array}{cc} T\; & =\left(1+H^{\prime }g+H^{\prime }gH^{\prime }g+...\right)H^{\prime }=H^{\prime }\left(1+gH^{\prime }+gH^{\prime }gH^{\prime }+...\right)\\ \; & =\frac{H^{\prime }}{1-H^{\prime }g}=\frac{H^{\prime }}{1-gH^{\prime }}\;. \end{array}$$

When we restrict ourselves to the normal case of a continuous energy spectrum consisting of extended eigenstates, for ϵ of real values belonging to such a spectrum, we define Green’s functions as follows:

(114a)g±(ϵ)=lims→±01ϵ−H⊖+is=lims→±0∑i|ψi∘〉〈ψi∘|ϵ−ϵi∘+is(114b)G±(ϵ)=lims→±01ϵ−H+is=lims→±0∑i|ψi〉〈ψi|ϵ−ϵi+is.

The Dirac’s bra and ket notation is particularly useful hereafter, as working with Green’s functions is greatly facilitated by introducing an abstract vector space [24].

Using the Sokhotski–Plemelj theorem, $\lim_{s\to \pm 0}\frac{1}{x+i\;s}=P\left[\frac{1}{x}\right]\mp i\;\pi \delta \left(x\right)$, the expressions of the Green’s functions can be more explicit by separating real and imaginary parts:

(115a)g±(ϵ)=∑iP[|ψi∘〉〈ψi∘|ϵ−ϵi∘]∓iπ∑iδ(ϵ−ϵi∘)|ψi∘〉〈ψi∘|(115b)G±(ϵ)=∑iP[|ψi〉〈ψiϵ−ϵi|]∓iπ∑iδ(ϵ−ϵi)|ψi〉〈ψi|,

where P is the Cauchy principal value, which is regarded as having the following property [25]:

(116) $$\begin{array}{c} P\left[x^{-1}\right]=\left\{\begin{array}{cc} 0 & \mathrm{when}\;\;x=0\;,\\ x^{-1} & \mathrm{when}\;\;x\neq 0\;. \end{array}\right. \end{array}$$

Assuming that ϵ belongs to the continuous spectrum of both H and $H^{⊖}$, the eigenfuctions for the initial and perturbed systems are related via the Lippman–Schwinger equation:

(117) $$\begin{array}{c} |\psi^{\pm }\rangle =|\psi^{\circ }\rangle +g^{\pm }\left(\epsilon \right)H^{\prime }|\psi^{\pm }\rangle =|\psi^{\circ }\rangle +G^{\pm }\left(\epsilon \right)H^{\prime }|\psi^{\circ }\rangle \;, \end{array}$$

where $|\psi_{i}^{\circ }\rangle$ is the general solution of $\left(\epsilon -H^{⊖}\right)|\psi_{i}^{\circ }\rangle =0$, equivalent to Equation ().

The Green’s function-based expression for the electronic band structure energy change due to a perturbation has been developed by using the Lloyd formula with the system described by $H^{⊖}$ as the reference, i.e., with the zero state ∘ as the starting point [26,27]. With our definition of the state ^★^, the Lloyd formula is given as follows:

(118) Δ★N(ϵ)=N★(ϵ)−N∘(ϵ)=±1πIm{TrlnT±(ϵ)},

where the trace symbol, Tr, denotes integration in both the position eigenvector space |r〉 and spin space. Therefore, the change in the band structure energy is estimated by inserting Equation (118) into Equation (108b) and calculating the transfer matrix elements, under the assumption that the band structure change is small ($\epsilon_{F}^{★}\approx \epsilon_{F}^{\circ }$):

(119) δ★Eb.s.≈∓1π∫−∞ϵF∘dϵIm{TrlnT±(ϵ)},

which forms the basis for the Lichtenstein formula [4,28].

We now proceed to find an alternative expression for Equation (119) that shows the equivalence of the results from the two methods, the Brillouin–Wigner perturbation method and the Green’s function perturbation method. We start this by expressing $\ln T^{\pm }$ in Equation (118) in terms of a series using Equation (113):

(120) $$\begin{array}{cc} \ln T\; & =\ln\;\frac{H^{\prime }}{1-H^{\prime }g}=\ln\;H^{\prime }-\;\ln\;\left(1-H^{\prime }g\right)=\ln\;H^{\prime }+\sum_{n=1}^{\infty }\frac{{\left(H^{\prime }g\right)}^{n}}{n}\;. \end{array}$$

After replacing *g* with $g^{+}$ (or, $g^{-}$) in Equation (115a), we rewrite ${\left(H^{\prime }g\right)}^{n}$ in the trace as a binomial expansion:

(121) Tr(H′g±)n=Tr(H′Re{g±}+iH′Im{g±})n=∑k=0n(i)knkTr(H′Im{g±})k(H′Re{g±})n−k.

For the second identity here, we used the fact that H′Im{g±}, and H′Re{g±} are not commutative, and yet the trace of the permutative multiplication terms are invariant [24]. From Equations (120) and (121), therefore, the imaginary part of $Tr\;\left[\ln T^{\pm }\right]$ can be rewritten as follows:

(122) Im{TrlnT±}=∑n=1∞1n∑k=1,oddn(−1)k−12nkTr(H′Im{g±})k(H′Re{g±})n−k.

Upon integration of Equation (122) over ϵ, the sum becomes significantly simpler, containing only the terms with k=1:

(123) ∫−∞ϵF∘dϵIm{TrlnT±}=∑n=1∞∫−∞ϵF∘dϵTr(H′Im{g±})(H′Re{g±})n−1.

This is because the integration causes any multiplication terms containing (H′Im{g±})k of *k* larger than 1 to have the null diagonal $H_{ii}^{\prime }$ matrix elements, owing to the property of the Dirac delta function in the expression for Im{g±} in Equation (115a).

This is illustrated by using the multiplication term n=2 and k=2 in the Tr[] in Equation (122). After inserting the imaginary part of $g^{\pm }$ of Equation (115a) in the term and integrating over ϵ, we find it becomes zero, with help of $H_{ii}^{\prime }=0$:

(124) ∫−∞ϵF∘dϵTr(H′Im{g±})2=(∓π)2∫−∞ϵF∘dϵ∑i,jδ(ϵ−ϵi∘)δ(ϵ−ϵj∘)TrH′|ψi∘〉〈ψi∘|H′|ψj∘〉〈ψj∘|=π2∑iocc.|〈ψi∘|H′|ψi∘〉|2=π2∑iocc.Hii′2=0.

In contrast, it can be shown that any terms having k=1 do not vanish. For example, with n=2 and k=1:

(125) ∫−∞ϵF∘dϵTr(H′Im{g±})(H′Re{g±})=∓π∫−∞ϵF∘dϵ∑i,jδ(ϵ−ϵi∘)Tr[H′|ψi∘〉〈ψi∘|H′P[|ψj∘〉〈ψj∘|ϵ−ϵj∘]]=∓π∑iocc.∑j≠i〈ψi∘|H′|ψj∘〉〈ψj∘|H′|ψi∘〉ϵi∘−ϵj∘=∓π∑iocc.∑j≠iHij′Hji′ϵi∘−ϵj∘.

After applying the same procedure to all the terms with k=1, Equation (123) is re-expressed explicitly in terms of the spinor interaction matrix elements:

(126) ∓1π∫−∞ϵF∘dϵIm{TrlnT±(ϵ)}=∑iocc.∑j≠iHij′Hji′ϵi∘−ϵj∘+∑k≠iHij′Hjk′Hki′(ϵi∘−ϵj∘)(ϵi∘−ϵk∘)+....

Insertion of Equation (126) into Equation (119) would lead to the expression for $\delta^{★}E_{e.s.}$ as a continuous sum:

(127) $$\delta^{★}E_{b.s.}\approx \sum_{i}^{occ.}\sum_{j\neq i}\left[\frac{H_{ij}^{\prime }H_{ji}^{\prime }}{\epsilon_{i}^{\circ }-\epsilon_{j}^{\circ }}+\sum_{k\neq i}\frac{H_{ij}^{\prime }H_{jk}^{\prime }H_{ki}^{\prime }}{\left(\epsilon_{i}^{\circ }-\epsilon_{j}^{\circ }\right)\left(\epsilon_{i}^{\circ }-\epsilon_{k}^{⊖}\right)}+...\right]\;,$$

which proves the equivalence between Equations (103) and (119).

### 2.3. Examples

While the analytical expressions of the energy terms derived so far are quite general, they can have much simpler expressions after imposing additional approximations specific to a given magnetic system. Here, we examine the two simplest magnetic systems at two opposite extremes; one with (active) magnetic orbitals in Mott–Hubbard magnetic insulators and the other with localized magnetic moments that interact indirectly only via (passive) conducting electrons (RKKY materials). Our previous work has utilized the first example as a simpler form of the magnetic transition metal dimer complexes [16].

#### 2.3.1. Magnetic Dimer Complexes

For the magnetic transition metal dimer complexes, $E_{\mathrm{sp}}^{\circ }$ and $\delta^{★}E_{e.s.}$ in the expression of $J_{\mathrm{ab}}$ in Equation (73) are of interest. The transition metal ions in the dimers may or may not be related by symmetry. Although a general description is available [29,30], for simplicity, we consider a transition metal dimer complex that contains only one unpaired electron on each of the two transition metal sites. Magnetic exchange interactions between the unpaired electrons are assumed to be well described by considering only the interactions between the two *d*-orbitals containing those unpaired electrons (active electron approximation).

**Figure 2:**
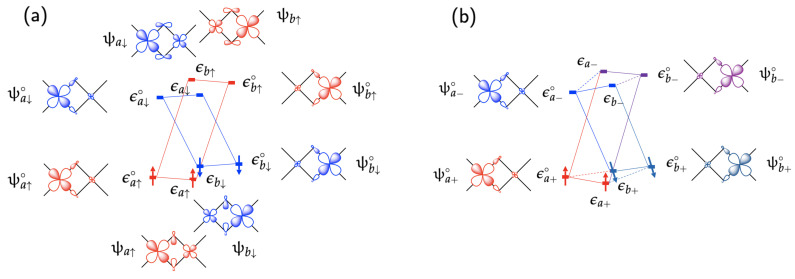
Schematic diagrams of spinor interactions in a simple transition metal dimer complex with two magnetic sites ***a*** on the left and ***b*** on the right with a single magnetic orbital on each site, (**a**) in the antiferromagnetic state and (**b**) in a general magnetic state with the spin directions angled at θ from each other.

Figure 2 illustrates the relationships among various expressions of spinors and energy terms by using a simple nearly symmetric transition metal dimer complex that contains one unpaired electron on each of the two transition metal sites a and b. There are four magnetic spinors for description of the magnetic interactions:

(128) $$\begin{array}{c} \psi_{a+}^{\circ }=\psi_{a}^{\circ }\omega_{a+}^{\circ }\;,\;\;\psi_{a-}^{\circ }=\psi_{a}^{\circ }\omega_{a-}^{\circ }\;,\;\;\psi_{b+}^{\circ }=\psi_{b}^{\circ }\omega_{b+}^{\circ }\;,\;\;\psi_{b-}^{\circ }=\psi_{b}^{\circ }\omega_{b-}^{\circ }\;, \end{array}$$

and their corresponding spin density vectors are given as follows:

(129) $$\begin{array}{c} {\vec{\rho }}_{a+}^{\;S\circ }=\rho_{a}^{\circ }u_{a+}^{\circ }\;,\;\;{\vec{\rho }}_{a-}^{\;S\circ }=\rho_{a}^{\circ }u_{a-}^{\circ }\;,\;\;{\vec{\rho }}_{b+}^{\;S\circ }=\rho_{b}^{\circ }u_{b+}^{\circ }\;,\;\;{\vec{\rho }}_{b-}^{\;S\circ }=\rho_{b}^{\circ }u_{b-}^{\circ }\;, \end{array}$$

where $\psi_{a}^{\circ }$ and $\psi_{a}^{\circ }$ are magnetic orbitals with their corresponding orbital electron densities, $\rho_{a}^{\circ }$ and $\rho_{b}^{\circ }$, respectively. The symbols $\omega_{i+}$ and $\omega_{i-}$ (i=a or *b*) denote a Kramers pair of unit spinors whose spin orientations are opposite to each other ($u_{i-}=-u_{i+}$). As a convention, we will designate + for the magnetic spinor with the lower energy in a Kramers pair and − for the one with the higher energy. In case the magnetic ions are coupled antiferromagnetically, as depicted in Figure 2a, the magnetic spinors and their spin density vectors can be re-expressed by setting +=↑ for the site a and +=↓ for the site b:

(130a)ψa↑∘=ψa∘ω↑,ψa↓∘=ψa∘ω↓,ψb↑∘=ψb∘ω↑,ψb↓∘=ψb∘ω↓,(130b)ρ→a↑S∘=ρa∘u↑,ρ→a↓S∘=ρa∘u↓,ρ→b↑S∘=ρb∘u↑,ρ→b↓S∘=ρb∘u↓.

Since only $\psi_{a↑}^{\circ }$ and $\psi_{b↓}^{\circ }$ are occupied by electrons for the antiferromagnetic state (*broken state*), the zeroth-order total spin density vector is given as follows:

(131) $$\begin{array}{c} {\vec{\rho }}_{\;S}^{\circ \mathrm{AF}}={\vec{\rho }}_{a↑}^{\;S\circ }+{\vec{\rho }}_{b↓}^{\;S\circ }=\rho_{a}^{\circ }u_{↑}+\rho_{b}^{\circ }u_{↓}\;. \end{array}$$

From Equations (42), (44), (80a), (82a), (130) and (131), we obtain the magnetic spinor energies,

(132a)ϵa↑∘≈εaa∘−12(Ua−Kab),ϵa↓∘≈εaa∘+12(Ua−Kab),(132b)ϵb↓∘≈εbb∘−12(Ub−Kab),ϵb↑∘≈εbb∘+12(Ub−Kab),

and the spin polarization energy of the antiferromagnetic zero state (Equation (69)) is as follows:

(133) $$\begin{array}{ccc} E_{\mathrm{sp}}^{\circ \mathrm{AF}} & \approx & -\frac{1}{4}\left(U_{a}+U_{b}-2K_{ab}\right)\;, \end{array}$$

where the self-orbital xc energies, $U_{i}$(i=a,b), and the inter-orbital xc energy, $K_{ab}$, are defined as follows:

(134a)Ui:=12∫∫ρ→iσS∘·Kxc(r,r′)·ρ→iσS∘(r)dr′dr≈LSD12∫κxc(ρi∘)2dr,(134b)Kab:=12∫∫ρ→aσS∘(r′)·Kxc(r,r′)·ρ→bσS∘(r)dr′dr≈LSD12∫κxc(ρa∘ρb∘)dr.

For the general case with the occupied magnetic spinors, $\psi_{a+}^{\circ }$ and $\psi_{b+}^{\circ }$, as shown in Figure 2b, the zeroth-order total spin density vector is expressed as follows:

(135) $$\begin{array}{c} {\vec{\rho }}_{S}^{\;\circ }={\vec{\rho }}_{a+}^{\;S\circ }+{\vec{\rho }}_{b+}^{\;S\circ }\;. \end{array}$$

The corresponding magnetic spinor energies are as follows:

(136a)ϵa+∘≈εaa∘−12(Ua+Kabua+∘·ub+∘),ϵa−∘≈εaa∘+12(Ua+Kabua+∘·ub+∘),(136b)ϵb+∘≈εbb∘−12(Ub+Kabua+∘·ub+∘),ϵb−∘≈εbb∘+12(Ub+Kabua+∘·ub+∘),

While the general expression of the spin polarization energy of the zero state for this magnetic dimer is as follows:

(137) $$\begin{array}{ccc} E_{\mathrm{sp}}^{\circ } & \approx & -\frac{1}{4}\left(U_{a}+U_{b}+2K_{ab}\;u_{a+}^{\circ }\cdot u_{b+}^{\circ }\right)\;. \end{array}$$

In calculation of $\delta^{★}E_{e.s.}$ for this dimer example, we will ignore the spin density-mediated inter-spinor interaction terms (i.e., ${\tilde{\Delta }}_{ji}^{S\circ }=0$), so we set $H_{ji}^{\prime }=\epsilon_{ji}^{\circ }$ for all the spinors in Equation (80b), which gives

(138) $$\begin{array}{ccc} \delta^{★}E_{e.s.} & \approx & \sum_{i}\;n_{i}^{\circ }\sum_{j\neq i}\frac{\epsilon_{ij}^{\circ }\epsilon_{ji}^{\circ }}{\epsilon_{i}^{\circ }-\epsilon_{j}^{\circ }}\;, \end{array}$$

to the second-order energy correction. This approximation has no clear justification but is customary in the literature. Treatment with the full expression for $H_{ji}^{\prime }=\epsilon_{ji}^{\circ }+{\tilde{\Delta }}_{ji}^{S\circ }$ will be given in future work. The numerators in the second-order terms in Equation (138) can be analyzed by first using the identity |ψj∘〉〈ψj∘|=12(1+uj∘·σ→)|ψj∘〉〈ψj∘| from the Pauli algebra and separating the spin-dependent components from the rest:

(139) $$\begin{array}{ccc} \epsilon_{ij}^{\circ }\epsilon_{ji}^{\circ } & = & \langle \psi_{i}^{\circ }|H^{\mathrm{nsp}}|\psi_{j}^{\circ }\rangle \langle \psi_{j}^{\circ }|H^{\mathrm{nsp}}|\psi_{i}^{\circ }\rangle =\frac{1}{2}\langle \psi_{i}^{\circ }|H^{\mathrm{nsp}}|\psi_{j}^{\circ }\rangle \langle \psi_{j}^{\circ }|H^{\mathrm{nsp}}|\psi_{i}^{\circ }\rangle \left[\omega_{i}^{\circ †}\left(1+u_{j}^{\circ }\cdot \vec{\sigma }\right)\omega_{i}^{\circ }\right]\\ & = & \frac{1}{2}{\left|\varepsilon_{ji}^{\circ }\right|}^{2}\left(1+u_{j}^{\circ }\cdot u_{i}^{\circ }\right)\;. \end{array}$$

By considering only the *net non-zero* interactions in Equation (138), which are between the occupied spinors and the unoccupied spinors, we find the following:

(140) $$\begin{array}{ccc} \delta^{★}E_{e.s.} & \approx & \frac{|\varepsilon_{ab}^{\circ }{|}^{2}\left(1+u_{a+}^{\circ }\cdot u_{b-}^{\circ }\right)}{2(\epsilon_{a+}^{\circ }-\epsilon_{b-}^{\circ })}+\frac{|\varepsilon_{ab}^{\circ }{|}^{2}\left(1+u_{b+}^{\circ }\cdot u_{a-}^{\circ }\right)}{2(\epsilon_{b+}^{\circ }-\epsilon_{a-}^{\circ })}\\ & = & -\frac{\Delta_{ab}^{2}\left(1-u_{a+}^{\circ }\cdot u_{b+}^{\circ }\right)}{4({\bar{U}}_{ab}+K_{ab}\;u_{a+}^{\circ }\cdot u_{b+}^{\circ }-w_{ab})}\;, \end{array}$$

after we use the first-order spinor energies in Equation (136) and define |εab∘|:=Δab2, U¯ab:=(Ua+Ub)2 and wab:=(εbb∘−εaa∘)2(U¯+Kabua+∘·ub+∘). Here, $\Delta_{ab}$ indicates the extent of spinless orbital interaction between the magnetic orbitals, and ${\bar{U}}_{ab}$ corresponds to the average on-site repulsion energy when the magnetic dimer is symmetric [14,16]. The term $w_{ab}$ reflects the effects of the energy difference of the magnetic orbitals in the nonmagnetic state, relative to the on-site repulsion energies. With Sa=Sb=12 and $u_{a+}^{\circ }\cdot u_{b+}^{\circ }=\cos\theta$, insertion of $E_{\mathrm{sp}}^{\circ }$ in Equation (137) and $\delta^{★}E_{e.s.}$ in Equation (140) into Equation (73) provides the expression for the magnetic exchange coupling constant for the simple dimer:

(141) $$\begin{array}{ccc} J_{\mathrm{ab}} & \approx & 2K_{ab}-\frac{\Delta_{ab}^{2}}{{\bar{U}}_{ab}+K_{ab}-w_{ab}}\;. \end{array}$$

The first and second terms in Equation (141) are ferromagnetic and antiferromagnetic components, respectively. We note that the ferromagnetic component originates fundamentally from $\Delta E_{\mathrm{tot}}^{\circ }$, the total energy correction in the first order, while the antiferromagnetic component is from spin-dependent interactions of the magnetic orbitals in the second order, ignoring the energy terms containing ${\vec{\rho }}_{S}^{\;\mathrm{ind}}$. According to Equation (134b), the inter-orbital xc energy $K_{ab}$ is large when the magnetic orbitals overlap strongly.

The expression in Equation (141) is slightly different from the one that we would expect from the energy difference between the ferromagnetic state and the antiferromagnetic state while being consistent with the prefactor in Equation (2) [16]:

(142) $$\begin{array}{ccc} J_{\mathrm{ab}} & = & \frac{1}{S_{a}S_{b}}\left(E_{\mathrm{tot}}^{\mathrm{AF}}-E_{\mathrm{tot}}^{F}\right)\approx 2K_{ab}-\frac{\Delta_{ab}^{2}}{{\bar{U}}_{ab}-K_{ab}-{\bar{w}}_{ab}}\;. \end{array}$$

This discrepancy is due to the fact that the antiferromagnetic term given in Equation (140) is not linearly related to $u_{a+}^{\circ }\cdot u_{b+}^{\circ }$ (or, cosθ).

For symmetric 12-dimers, an analogous expression has been obtained by explicitly analyzing the energy of the triplet state and the interaction between the ground singlet state and the excited charge transfer singlet state, using weakly interacting orthonormal magnetic orbitals to form the molecular orbitals for construction of Slater determinants [14,15]:

(143) $$\begin{array}{ccc} J_{\mathrm{ab}} & \approx & 2K_{ab}-\frac{\Delta_{ab}^{2}}{J_{ab}-j_{ab}}\;, \end{array}$$

where we employ the following two-electron integrals:

(144a)Jab:=〈ψa∘(1)ψa∘(2)|r12−1|ψa∘(1)ψa∘(2)〉,(144b)jab:=〈ψa∘(1)ψb∘(2)|r12−1|ψa∘(1)ψb∘(2)〉,(144c)Kab:=〈ψa∘(1)ψb∘(2)|r12−1|ψa∘(2)ψb∘(1)〉.

The exchange integral between the two *real-function* magnetic orbitals $\psi_{a}^{\circ }$ and $\psi_{b}^{\circ }$ can be interpreted as the electron repulsion energy between electrons 1 and 2 that have the same overlap density $\rho_{ab}^{\circ }=\psi_{a}^{\circ }\psi_{b}^{\circ }$ [15]:

(145) $$\begin{array}{c} K_{ab}=\int \frac{\rho_{ab}^{\circ }\left(1\right)\rho_{ab}^{\circ }\left(2\right)}{r_{12}}dr\;. \end{array}$$

When the overlap is large between the two *orthonormal* magnetic orbitals, the ferromagnetic coupling interaction is strong between the unpaired electrons in the orbitals. The corresponding quantity $K_{ab}$ in Equation (134b) evinces the same information, as it delineates the extent of the overlap between the electron densities of $\psi_{a}^{\circ }$ and $\psi_{b}^{\circ }$, weighted with the spin stiffness function. Meanwhile, the second-order terms in Equations (141), (142) and (143) measure the extent of the antiferromagnetic interactions of the magnetic orbitals that are due to their mixing and thus delocalization among the magnetic sites. Both $U_{ab}$ in Equation (134a) and $J_{ab}$ in Equation (144a) correspond to the on-site repulsion energy *U*, which measures the Coulombic repulsion energy of the two electrons in the same magnetic orbital. The delocalization of the electrons in the magnetic orbitals would be strong when $\Delta_{ab}$ is large and the on-site repulsion is small.

#### 2.3.2. RKKY Exchange Interactions Between Point Magnetic Moments

**Figure 3:**
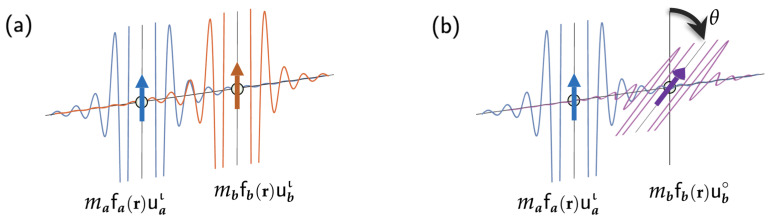
Schematic diagrams of free-electron Friedel oscillations induced by two local magnetic sites ***a*** and ***b*** with (**a**) a ferromagnetic spin alignment and (**b**) a spin alignment resulting from a small spin rotation at the site ***b***.

For the RKKY-type exchange interactions, the perturbation occurs through the spin reorientation of localized magnetic moments without a change in the spacial part. The change is also negligible in the spacial part of of the spinors for the mediating conducting electrons. Figure 3 shows a simple RKKY system in which two magnetic sites are far apart from each other and interact ferromagnetically through free electrons. The total spin density ${\vec{\rho }}_{S}^{\;\iota }$ is the sum of the point-spin-densities at the two sites ***a*** and ***b*** at positions a and b, respectively, and the net spin densities among the free conducting electrons induced by the two magnetic sites:

(146) $$\begin{array}{ccc} {\vec{\rho }}_{S}^{\;\iota }\left(r\right) & = & m_{a}\left(\delta_{a}\left(r\right)+f_{a}\left(r\right)\right)u_{a}^{\iota }+m_{b}\left(\delta_{b}\left(r\right)+f_{b}\left(r\right)\right)u_{b}^{\iota }\;, \end{array}$$

where *m* and $u^{\iota }$ are the magnetic momentum and spin-orientation unit vector of the localized sites, respectively. $\delta_{a}\left(r\right)$ and $\delta_{b}\left(r\right)$ are the Dirac delta functions centered at the positions a and b, describing the localized spin densities located at the sites, while $f_{a}\left(r\right)$ and $f_{b}\left(r\right)$ are proportional to Friedel functions centered at the two sites. The function $f_{a}\left(r\right)$ describes the spin density induced among the free electrons by the magnetic site a, and $f_{b}\left(r\right)$ caused by the site b [17]. The localized magnetic orbitals on the magnetic sites keep their shape the same in all magnetic states, regardless of their spin orientations. The spin components of the spinors of the free electrons are r-dependent. In the initial state ι, the spinors of the conducting electrons are expressed as follows:

(147) $$\begin{array}{ccc} \phi_{i}^{\iota }\left(r\right) & = & \phi_{i}^{\iota }\left(r\right)\omega_{i}^{\iota }\left(r\right)\;. \end{array}$$

The summation of the spin densities of the occupied $\phi_{i}^{\iota }$s gives the following:

(148) $$\begin{array}{ccc} \int_{-\infty }^{\epsilon_{F}^{\iota }}\phi_{i}^{\iota †}\vec{\sigma }\phi_{i}^{\iota }\;d\epsilon & = & m_{a}f_{a}\left(r\right)u_{a}^{\iota }+m_{b}f_{b}\left(r\right)u_{b}^{\iota }\;. \end{array}$$

In the ferromagnetic arrangement of the magnetic moments, $u_{a}^{\iota }$ and $u_{b}^{\iota }$ are parallel to each other.

Imagine that the system is transitioned to a different magnetic state, shown in Figure 3b, in which the magnetic site b has $u_{b}^{\circ }$ that is oriented at an angle θ with respect to $u_{a}^{\iota }$. That is,

(149) $$\begin{array}{c} u_{a}^{\iota }\cdot u_{b}^{\circ }=\cos\theta \;, \end{array}$$

and its spin density is to a first approximation given as follows:

(150) $$\begin{array}{ccc} {\vec{\rho }}_{S}\left(r\right) & = & m_{a}\left(\delta_{a}\left(r\right)+f_{a}\left(r\right)\right)u_{a}^{\iota }+m_{b}\left(\delta_{b}\left(r\right)+f_{b}\left(r\right)\right)u_{b}^{\circ }\;. \end{array}$$

This approximation is equivalent to say that the spinors of this magnetic state have the same spatial components as the initial spinors, but with changed spin components:

(151) $$\begin{array}{ccc} \psi_{i}\left(r\right) & = & \phi_{i}^{\iota }\left(r\right)\omega_{i}\left(r\right)\;, \end{array}$$

so that the summation of the spin densities of the occupied $\psi_{i}$s of the conducting electrons gives the following:

(152) $$\begin{array}{ccc} \int_{-\infty }^{\epsilon_{F}}\psi_{i}^{†}\vec{\sigma }\psi_{i}\;d\epsilon & = & m_{b}f_{b}\left(r\right)u_{b}^{\circ }\;. \end{array}$$

Since the spatial part of the spinors does not change during the change in the magnetic state, the total energy change comes solely from the spin polarization energies (Equation (89)):

(153) $$\begin{array}{ccc} \Delta E_{\mathrm{tot}} & \approx & \Delta E_{\mathrm{sp}}=E_{\mathrm{sp}}\left[\rho ,{\vec{\rho }}_{S}\right]-E_{\mathrm{sp}}\left[\rho ,{\vec{\rho }}_{S}^{\;\iota }\right]\\ & \overset{\mathrm{LSD}}{\approx } & -\frac{1}{2}m_{a}m_{b}\left(\kappa_{\mathrm{xc}}\left(b\right)f_{a}\left(b\right)+\kappa_{\mathrm{xc}}\left(a\right)f_{b}\left(a\right)\right)u_{a}^{\iota }\cdot \left(u_{b}^{\circ }-u_{b}^{\iota }\right)\;. \end{array}$$

For the last approximation, we inserted ${\vec{\rho }}_{S}$ in Equation (150) and ${\vec{\rho }}_{S}^{\;\iota }$ in Equation (146) into Equation (30) within the LSD approximation, and we performed the integration using the properties of the Dirac delta function after ignoring the terms in the second order of the f(r) functions. Thus, with Equation (2), as well as with Equation (149) and m=2S, we find the following:

(154) $$\begin{array}{ccc} J_{\mathrm{ab}}^{\mathrm{RKKY}} & \overset{\mathrm{LSD}}{\approx } & 2(\kappa_{\mathrm{xc}}\left(b\right)f_{a}\left(b\right)+\kappa_{\mathrm{xc}}\left(a\right)f_{b}\left(a\right))\;. \end{array}$$

In Equation (154), $\kappa_{\mathrm{xc}}\left(b\right)f_{a}\left(b\right)$ represents the interaction between the magnetic site b and the spin density induced by the site a, while $\kappa_{\mathrm{xc}}\left(a\right)f_{b}\left(a\right)$ represents the interaction between site a and the spin density induced by site b. Since the spin stiffness function $\kappa_{\mathrm{xc}}\left(r\right)$ is always positive, the RKKY system favors a ferromagnetic state when $f_{a}\left(b\right)$ and $f_{b}\left(a\right)$ have a positive value. We note that we can obtain Equation (154) by using the expression of $J_{\mathrm{ab}}$ in Equation (32), where the first term in the parentheses is constant and can be omitted in this case.

Alternatively, we now use the perturbational approach developed in the previous sections, especially Equation (105), for the expression of $J_{\mathrm{ab}}$, and we try to obtain the result of Equation (154). Naturally, the perturbation that would bring the initial magnetic state in Figure 3a to the final state in Figure 3b is the one that rotates $u_{b}^{\iota }$ to $u_{b}^{\circ }$ on the magnetic site b to give Equation (149). The resulting spin density change is then given as follows:

(155) $$\begin{array}{ccc} \delta {\vec{\rho }}_{S}^{\;\circ }\left(r\right) & = & m_{b}\delta_{b}\left(r\right)\left(u_{b}^{\circ }-u_{b}^{\iota }\right)\;, \end{array}$$

and the spin density of the zero state as

(156) $$\begin{array}{ccc} {\vec{\rho }}_{S}^{\;\circ }\left(r\right) & = & m_{a}\left(\delta_{a}\left(r\right)+f_{a}\left(r\right)\right)u_{a}^{\iota }+m_{b}\left(\delta_{b}\left(r\right)u_{b}^{\circ }+f_{b}\left(r\right)u_{b}^{\iota }\right)\;. \end{array}$$

In the subsequent spin relaxation process, among the conducting electrons, the change in the spin orientation at the site b would then reorient the spin direction associated with the $f_{b}\left(r\right)$ function so that its spin direction becomes parallel to $u_{b}^{\circ }$. The mathematical treatment of the spin relaxation is not straightforward in this case, yet we can still directly find the resulting induced spin density as the difference between ${\vec{\rho }}_{S}$ in Equation (150) and ${\vec{\rho }}_{S}^{\;\circ }$ in Equation (156):

(157) $$\begin{array}{ccc} {\vec{\rho }}_{S}^{\;\mathrm{ind}}\left(r\right) & = & {\vec{\rho }}_{S}\left(r\right)-{\vec{\rho }}_{S}^{\;\circ }\left(r\right)=m_{b}f_{b}\left(r\right)\left(u_{b}^{\circ }-u_{b}^{\iota }\right)\;. \end{array}$$

What follows is the calculation of $J_{\mathrm{ab}}$ by utilizing its expression in Equation (73). First, $\Delta E_{\mathrm{sp}}^{\circ }$ is obtained as follows:

(158) $$\begin{array}{ccc} \Delta E_{\mathrm{sp}}^{\circ } & \overset{\mathrm{LSD}}{\approx } & -\frac{1}{2}m_{b}^{2}\kappa_{\mathrm{xc}}\left(b\right)f_{b}\left(b\right)u_{b}^{\iota }\cdot \left(u_{b}^{\circ }-u_{b}^{\iota }\right)-\frac{1}{2}m_{a}m_{b}\kappa_{\mathrm{xc}}\left(b\right)f_{a}\left(b\right)u_{a}^{\iota }\cdot \left(u_{b}^{\circ }-u_{b}^{\iota }\right)\;, \end{array}$$

after inserting Equation (156) into Equation (30) for ${\vec{\rho }}_{S}^{\;\circ }$ within the LSD approximation, performing the integration using the properties of the Dirac delta function and ignoring the terms in the second order of the f(r) functions. Similarly, we calculate $\delta^{★}E_{e.s.}$ by inserting ${\vec{\rho }}_{S}^{\;\circ }$ into Equation (156) and ${\vec{\rho }}_{S}^{\;\mathrm{ind}}$ of Equation (157) and Equation (91) for passive electrons:

(159) $$\begin{array}{ccc} \delta^{★}E_{b.s.} & \overset{\mathrm{LSD}}{\approx } & -\frac{1}{2}m_{b}^{2}\kappa_{\mathrm{xc}}\left(b\right)\;f_{b}\left(b\right)\;u_{b}^{\circ }\cdot \left(u_{b}^{\circ }-u_{b}^{\iota }\right)-\frac{1}{2}m_{a}m_{b}\kappa_{\mathrm{xc}}\left(a\right)\;f_{b}\left(a\right)\;u_{a}^{\iota }\cdot \left(u_{b}^{\circ }-u_{b}^{\iota }\right)\;. \end{array}$$

After inserting Equations (158) and (159) into Equation (105), and utilizing Equation (149) and m=2S, we finally find the same expression for $J_{\mathrm{ab}}^{\mathrm{RKKY}}$ in Equation (154). Equivalently, we may use Equation (88) for the calculation. A closer examination indicates that the first terms in Equations (158) and (159) are dominant in each energy component because $f_{b}\left(b\right)\gg f_{b}\left(a\right)$, but they cancel out in the summation in Equation (105).

Although not shown graphically, we can consider an alternative perturbation which rotates $u_{a}^{\iota }$ to $u_{a}^{\circ }$ on the magnetic site a and $u_{b}^{\iota }$ to $u_{b}^{\circ }$ on the magnetic site b in such a way that

(160) $$\begin{array}{c} u_{a}^{\circ }\cdot u_{b}^{\circ }=\cos\theta \;. \end{array}$$

The spin density change that represents this perturbation is given as follows:

(161) $$\begin{array}{ccc} \delta {\vec{\rho }}_{S}^{\;\circ }\left(r\right) & = & m_{a}\delta_{a}\left(r\right)\left(u_{a}^{\circ }-u_{a}^{\iota }\right)+m_{b}\delta_{b}\left(r\right)\left(u_{b}^{\circ }-u_{b}^{\iota }\right)\;, \end{array}$$

And the spin density of the zero state is as follows:

(162) $$\begin{array}{ccc} {\vec{\rho }}_{S}^{\;\circ }\left(r\right) & = & m_{a}\left(\delta_{a}\left(r\right)u_{a}^{\circ }+f_{a}\left(r\right)u_{a}^{\iota }\right)+m_{b}\left(\delta_{b}\left(r\right)u_{b}^{\circ }+f_{b}\left(r\right)u_{b}^{\iota }\right)\;. \end{array}$$

With these, the corresponding induced spin density is obtained as follows:

(163) $$\begin{array}{ccc} {\vec{\rho }}_{S}^{\;\mathrm{ind}}\left(r\right) & = & {\vec{\rho }}_{S}\left(r\right)-{\vec{\rho }}_{S}^{\;\circ }\left(r\right)=m_{a}f_{a}\left(r\right)\left(u_{a}^{\circ }-u_{a}^{\iota }\right)+m_{b}f_{b}\left(r\right)\left(u_{b}^{\circ }-u_{b}^{\iota }\right)\;. \end{array}$$

By following the same procedure above, we find the following:

(164) $$\begin{array}{ccc} \Delta E_{\mathrm{sp}}^{\circ } & \overset{\mathrm{LSD}}{\approx } & -\frac{1}{2}m_{a}^{2}\kappa_{\mathrm{xc}}\left(a\right)f_{a}\left(a\right)u_{a}^{\iota }\cdot \left(u_{a}^{\circ }-u_{a}^{\iota }\right)-\frac{1}{2}m_{b}^{2}\kappa_{\mathrm{xc}}\left(b\right)f_{b}\left(b\right)u_{b}^{\iota }\cdot \left(u_{b}^{\circ }-u_{b}^{\iota }\right)\\ & & -\frac{1}{2}m_{a}m_{b}\left(\kappa_{\mathrm{xc}}\left(a\right)f_{b}\left(a\right)u_{a}^{\circ }\cdot u_{b}^{\iota }+\kappa_{\mathrm{xc}}\left(b\right)f_{a}\left(b\right)u_{a}^{\iota }\cdot u_{b}^{\circ }\right)\;, \end{array}$$

and

(165) $$\begin{array}{ccc} \delta^{★}E_{b.s.} & \overset{\mathrm{LSD}}{\approx } & -\frac{1}{2}m_{a}^{2}\kappa_{\mathrm{xc}}\left(a\right)f_{a}\left(a\right)u_{a}^{\circ }\cdot \left(u_{a}^{\circ }-u_{a}^{\iota }\right)-\frac{1}{2}m_{b}^{2}\kappa_{\mathrm{xc}}\left(b\right)f_{b}\left(b\right)u_{b}^{\circ }\cdot \left(u_{b}^{\circ }-u_{b}^{\iota }\right)\\ & & \;\;-\frac{1}{2}m_{a}m_{b}\left[\kappa_{\mathrm{xc}}\left(a\right)f_{b}\left(a\right)u_{a}^{\circ }\cdot \left(u_{b}^{\circ }-u_{b}^{\iota }\right)+\kappa_{\mathrm{xc}}\left(b\right)f_{a}\left(b\right)u_{b}^{\circ }\cdot \left(u_{a}^{\circ }-u_{a}^{\iota }\right)\right]\;. \end{array}$$

Thus, with Equations (164) and (165), as well as Equation (160) and m=2S, the same expression of $J_{\mathrm{ab}}$ in Equation (154) emerges, as in the previous choice of the perturbation of Equation (155). It is observed that with our choices of $\delta {\vec{\rho }}_{S}^{\;\circ }$ so far in Equations (155) and (161), neither the $\Delta E_{\mathrm{sp}}^{\circ }$ (or equivalently $\Delta E_{\mathrm{tot}}^{\circ }$) or $\delta^{★}E_{b.s.}$ terms provide the correct expression for $J_{\mathrm{ab}}^{\mathrm{RKKY}}$ by itself; only their sum does. Furthermore, $\delta^{★}E_{b.s.}$ will contain both $f_{a}\left(b\right)$ and $f_{b}\left(a\right)$ interaction terms only when the spin polarization is perturbed on both magnetic sites.

Based on these observations, we try to devise a way that $\Delta E_{\mathrm{sp}}^{\circ }$ vanishes and $\delta^{★}E_{b.s.}$ alone would allow for the calculation of the magnetic exchange coupling constants. For this, we choose another form of the spin polarization perturbation:

(166) $$\begin{array}{ccc} \delta {\vec{\rho }}_{S}^{\;\circ \;\mathrm{MT}}\left(r\right) & := & \delta_{a}\left(r\right)\left(m_{a}+m_{a}f_{a}\left(r\right)+m_{b}f_{b}\left(r\right)\right)\left(u_{a}^{\circ }-u_{a}^{\iota }\right)\\ & \; & \;\;+\delta_{b}\left(r\right)\left(m_{b}+m_{a}f_{a}\left(r\right)+m_{b}f_{b}\left(r\right)\right)\left(u_{b}^{\circ }-u_{b}^{\iota }\right)\;, \end{array}$$

where $u_{a}^{\circ }$ and $u_{b}^{\circ }$ are related *via* Equation (149), for the zero-state defined by the following:

(167) $$\begin{array}{ccc} {\vec{\rho }}_{S}^{\;\circ \;\mathrm{MT}}\left(r\right) & = & {\vec{\rho }}_{S}^{\;\iota }\left(r\right)+\delta {\vec{\rho }}_{S}^{\;\circ \;\mathrm{MT}}\left(r\right)\;. \end{array}$$

The perturbation is constructed in such a way that it reorients *all* the spin density components *at the position* of each site to a new direction, $u_{a}^{\circ }$ or $u_{b}^{\circ }$. That is, on the very site a, the reoriented spin density components include the localized magnetic moment at the site a ($m_{a}\delta_{a}\left(r\right)$) and both of the spin densities of the conducting electrons induced by the site a ($m_{a}\delta_{a}\left(r\right)f_{a}\left(r\right)$) and by the site b ($m_{b}\delta_{a}\left(r\right)f_{b}\left(r\right)$). The same goes for the site b. This insures that the spin polarization energy is the same before and after the perturbation *within the LSD approximation*:

(168) $$\begin{array}{ccc} \Delta E_{\mathrm{sp}}^{\circ \;\mathrm{MT}} & \overset{\mathrm{LSD}}{\approx } & 0\;. \end{array}$$

Meanwhile, from Equations (150) and (167), the corresponding induced spin density is obtained as follows:

(169) $$\begin{array}{ccc} {{\vec{\rho }}_{S}}^{\;\mathrm{ind}\;\mathrm{MT}}\left(r\right) & = & m_{a}f_{a}\left(r\right)\left[\left(1-\delta_{a}\left(r\right)-\delta_{b}\left(r\right)\right)\left(u_{a}^{\circ }-u_{a}^{\iota }\right)+\delta_{b}\left(r\right)\left(u_{a}^{\circ }-u_{b}^{\circ }\right)\right]\\ & \; & \;\;+m_{b}f_{b}\left(r\right)\left[\left(1-\delta_{a}\left(r\right)-\delta_{b}\left(r\right)\right)\left(u_{b}^{\circ }-u_{b}^{\iota }\right)+\delta_{a}\left(r\right)\left(u_{b}^{\circ }-u_{a}^{\circ }\right)\right]\;. \end{array}$$

By using Equation (91) for the energy band system, we find the following:

(170) $$\begin{array}{ccc} \delta^{★}E_{b.s.}^{\;\mathrm{MT}} & \overset{\mathrm{LSD}}{\approx } & -\frac{1}{2}\int \kappa_{\mathrm{xc}}\;{\vec{\rho }}_{S}^{\;\circ \;\mathrm{MT}}\left(r\right)\cdot {\vec{\rho }}_{S}^{\;\mathrm{ind}\;\mathrm{MT}}\left(r\right)\;dr\\ & \approx & -\frac{1}{2}m_{a}m_{b}\left[\kappa_{\mathrm{xc}}\left(b\right)f_{a}\left(b\right)u_{b}^{\circ }\cdot \left(u_{a}^{\circ }-u_{b}^{\circ }\right)+\kappa_{\mathrm{xc}}\left(a\right)f_{b}\left(a\right)u_{a}^{\circ }\cdot \left(u_{b}^{\circ }-u_{a}^{\circ }\right)\right]\\ & = & -\frac{1}{2}m_{a}m_{b}\left(\kappa_{\mathrm{xc}}\left(b\right)f_{a}\left(b\right)+\kappa_{\mathrm{xc}}\left(a\right)f_{b}\left(a\right)\right)\left(u_{a}^{\circ }\cdot u_{b}^{\circ }-1\right)\;. \end{array}$$

These results show that for RKKY systems, judiciously chosen perturbations can lead to the following:

(171) $$\begin{array}{ccc} J_{\mathrm{ab}}^{\mathrm{MT}} & = & -\frac{1}{S_{a}S_{b}}{\left[\frac{\partial^{2}\;\delta^{★}E_{b.s.}^{\;\mathrm{MT}}}{\partial \theta^{2}}\right]}_{0}\;, \end{array}$$

and insertion of $\delta^{★}E_{b.s.}^{\;\mathrm{MT}}$ in Equation (170) into Equation (171) would lead to the expression in Equation (154). A more general expression for the total energy change than the one from the point-atom model used for Equation (170) would be the foundation of the Lichtenstein’s recipe for calculation of magnetic exchange coupling constants for metals with the Lichtenstein formula in Equation (119). That is, to utilize the Lichtenstein formula, we would devise a perturbation of the nature of Equation (166), so that the energy component $\Delta E_{\mathrm{sp}}^{\circ }$ would vanish in Equation (105). Indeed the same type of perturbation, i.e., the θ2 rotations on both magnetic sites with opposite rotation directions, was devised in the original work when deriving the Lichtenstein formula [4].

The essentials of the form of the perturbation in Equation (166) can be realized in the electronic band structure calculation methods, such as LMTO, KKR-ASA, and LAPW, where spin polarization within atomic regions can be separated out mathematically from the inter-atomic regions within the formalism based on the muffin-tin (MT) approximation [4,28]. However, this form of the perturbation is reasonable only when the magnetic sites are far apart from each other and their localized spinors do not overlap, unlike in the case of transition metal complexes (Figure 2) or Mott–Hubbard insulators. This is because for those compounds, the potential energy component (spin polarization energy component) of the magnetic exchange coupling constant comes from the *overlap* of neighboring magnetic orbitals at each magnetic atom. The perturbation in Equation (166) does not account for the differences among different spin alignments (i.e., different magnetic states) in the spin polarization energy term of the zero state at the magnetic centers. Indeed, this is what is expected for the muffin tin approximation, as it places *non-overlapping spheres* that are centered on the atomic positions, and within these regions, the effective potential is approximated to be spherically symmetric around the given nucleus. Those non-overlapping spheres might be considered, and a extended version of the Dirac delta functions employed in Equation (166).

Rigorous analysis of the terms in Equation (126) in conjunction with the general expression for the magnetic exchange coupling constant requires an extensive use of the Pauli algebra, which will be published separately. It is mentioned that the resulting expression from the second-order terms in Equation (126) corresponds to the Lichtenstein formula for molecules reported in the literature and re-expressed here in accordance with the definitions in this work, after setting $\varepsilon_{ji}^{\circ }$ in $H_{ji}^{\prime }$ to be zero for all spinors in using Equations (171), (119), and (127) [5]:

(172a)JABGreen=12π∫−∞ϵF∘dϵIm{TrV^AT↑ABV^BT↓BA}(172b)≈−12∑iocc.∑j〈ψiα|V^B|ψjβ〉〈ψjβ|V^A|ψiα〉ϵiα−ϵjβ+∑jocc.∑i〈ψjβ|V^A|ψiα〉〈ψiα|V^B|ψjβ〉ϵjβ−ϵiα.

The first summation in the bracket in Equation (172b) represents the interaction between the spin density induced by site *A* and the localized spin density at site *B*, while the second summation is the interaction between the spin density induced by site *B* and the localized spin density at site *A*. The type of the spinor interactions that Equation (172) can describe is indirect interactions between two magnetic sites via formation of induced spin density. The explicit form of the indirect interaction shown in Equation (172) is in the second order of perturbation in spinor energies. Application of Equation (172) to transition metal dimer complexes would give a different, if not erroneous, result in comparison to Equation (141). This may explain recent findings that the Green’s function approach acceptably reproduces broken-symmetry energy difference couplings for weaker dinuclear couplings [12].

It is not clear how the same limitation manifests in implementing the Lichtenstein formula in the electronic band structure calculation methods for extended structure systems because of the variety in the approximate treatments of the wave functions in the atomic regions. Nonetheless, a recent plane wave implementation of the magnetic force theorem recognized the first-order energy term similar to ${\tilde{\Delta }}_{ii}^{S\circ }$ in Equation (82a) as an essential component of *J*, while their second-order terms resembled the Lichtenstein formula [31]. These energy terms were called longitudinal and transverse contributions, respectively, in their work. Unlike the KKR and related methods, this formulation allowed one to define arbitrary magnetic sites localized to predefined spatial regions, hence rendering the problem of finding Heisenberg parameters independent of any orbital decomposition of the problem. The calculated Heisenberg parameters were robust towards changes in the definition of magnetic sites.

## 3. Concluding Remarks

This work provides a detailed perturbational analysis of the energy components that are involved in magnetic exchange interactions in molecules and solids by using spinors as eigenfunctions within density functional formalism, which leads to a general formula for magnetic exchange coupling constants as given in Equation (73). Different magnetic states are defined by their spin densities with negligible changes in total electron density. Their energy differences are expressed in terms of the spin polarization energies and the spinor energy changes based on the magnetic force theorem. Detailed analysis of the energy terms involved in the magnetic force theorem and of a simple RKKY model indicates that the foundation of the Lichtenstein formula is the proposition that different magnetic states, e.g., ferromagnetic and antiferromagnetic states, are distinguished by the spin density differences only in the interatomic region, leading to the principal formula for the magnetic exchange coupling constants given in Equation (171). It is also shown that in general, the magnetic force theorem alone does not properly provide the total energy difference between magnetic states because it does not contain the first-order energy correction terms upon perturbation. The first-order terms describe direct interactions between transition metal ions and thus represent the well-known potential energy component (ferromagnetic component) in Anderson’s model for the magnetic exchange interactions of transition metal compounds as a consequence of the overlap of magnetic orbitals. These first-order terms are missing in the Lichtenstein formula for magnetic exchange constants, and it is concluded that the formula is not suitable to be directly applied for transition metal complexes and magnetic insulators when the magnetic orbitals overlap.

It is noted that this work does not provide any new recipe for calculation of the magnetic exchange coupling constants but rather clarifies the possible confusion in using the known recipes by providing rigorous mathematical analysis. It is hard to say how the missing components of the first order affect the actual computational results, because the computational methods have their own approximations in describing the potentials and wavefunctions in the inner region of atoms (in contrast to the interatomic region) which influence the first-order terms most. Further studies are required with well-devised computational schemes that can extract the first-order energy terms from the results. In any event, the magnetic spinor approach and the application of Pauli algebra is novel in this work in describing the interactions of localized magnetic orbitals that are given by the familiar scalar product of the spin vectors of the magnetic sites in the HDVV Hamiltonian. The impetus of this work was the author’s need to provide a firm ground to the magnetic force theorem while he was working on the spinor-based perturbational analysis of the effect of spin–orbit coupling on magnetic exchange coupling within density functional theory. The work will be published separately in the hope of providing some insights in understanding the magnetic interactions in complex systems where spin directions are neither parallel nor antiparallel.

## Data Availability

Data are contained within the article.
